# Correction: What Do We Learn from Spheroid Culture Systems? Insights from Tumorspheres Derived from Primary Colon Cancer Tissue

**DOI:** 10.1371/journal.pone.0150179

**Published:** 2016-02-29

**Authors:** The PLOS ONE Staff

## Notice of Republication

This article was republished on February 11, 2016, to correct figure readability errors in the PDF that were introduced during the typesetting process. The publisher apologizes for the errors. Please download this article again to view the correct version. The originally published, uncorrected article and the republished, corrected articles are provided here for reference.

## Supporting Information

S1 File Originally published, uncorrected article. (PDF)

S2 File Republished, corrected article. (PDF)
