# Supplementary figures and images for: What Do We Learn from Spheroid Culture Systems? Insights from Tumorspheres Derived from Primary Colon Cancer Tissue
Source: PLoS One. 2016 Jan 8;11(1):e0146052. doi: 10.1371/journal.pone.0146052 (PMC4706382; doi:10.1371/journal.pone.0146052)

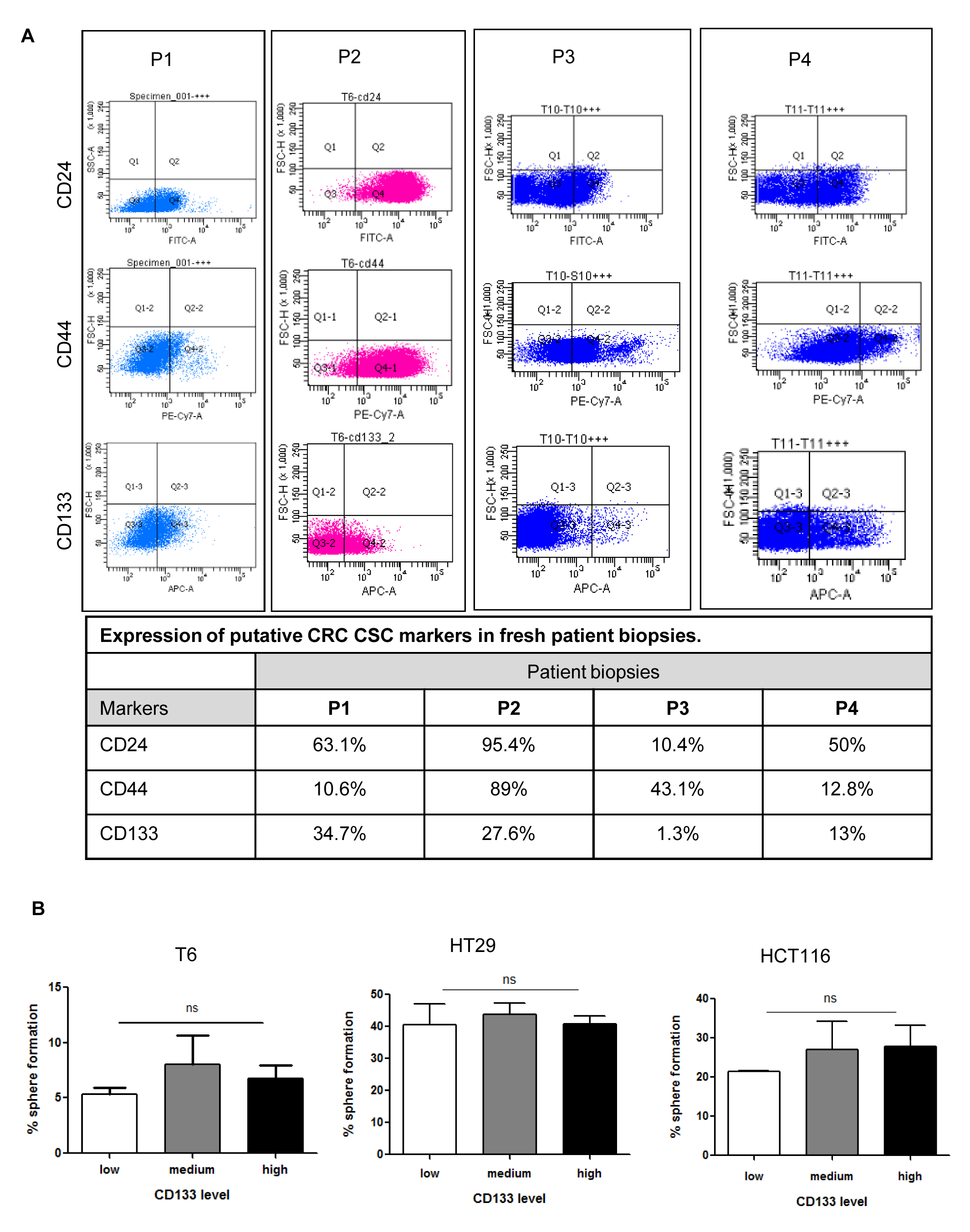

Supplement: S1 Fig — (A) Flow cytometry analysis of surface markers CD24, CD44 and CD133 in fresh resected tumor tissues from 4 different patients. (B) CD133 does not correlate with enhanced sphere-forming capacity. SC were sorted according to low, medium and high expression of the surface marker CD133, followed by a single cell sphere formation assay for CRC cell lines and T6 SC, data are presented as mean ± SD, ns = not significant. Representative figure from 2 independent experiments for HT29 and HCT116 while for T6 one single experiment was performed. (TIF) [file pone.0146052.s001.tif]

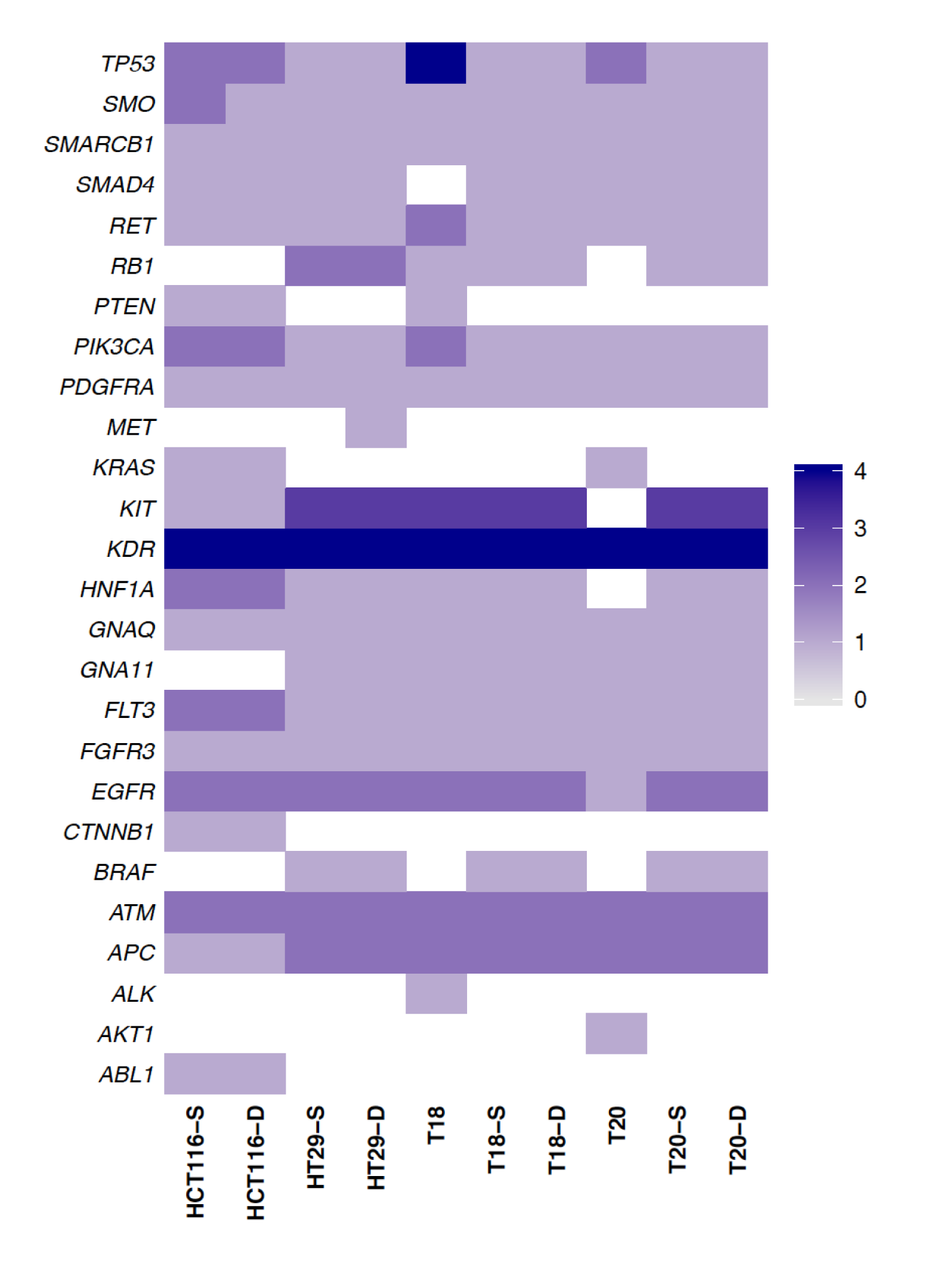

Supplement: S2 Fig — Several CRC-relevant mutations are shown for primary tumors, primary spheroid (S) and differentiated (D) cultures for T18 and T20 and for differentiated and spheroid cultures derived from cancer cell lines HCT116 and HT29. For ethical purposes we were not able to include the mutational profile for patient T6. Top mutations were assessed using the TruSeq Amplicon-Cancer panel (TSACP), depth > 1000, variant frequency > 0.05. Scale bar represents the number of mutations detected per gene. All mutations found in SC were also present in the differentiated counterpart. (TIF) [file pone.0146052.s002.tif]

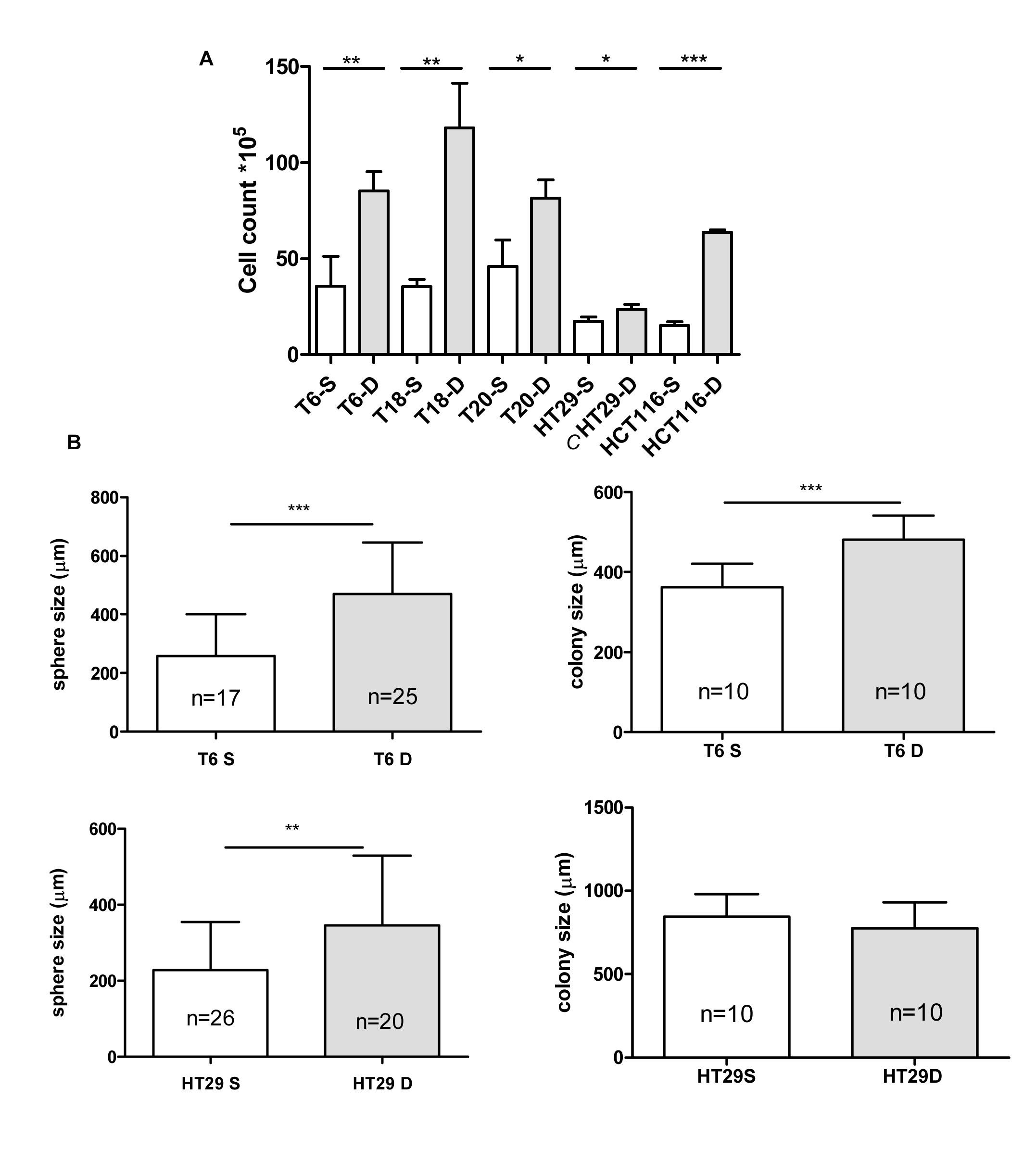

Supplement: S3 Fig — (A) Cell count observed after 5 days of culture for 100 000 cells initially plated. Representative figure of 2 independent experiments. (B) Spheres of differentiated cultures are bigger than spheres derived from SC. (C) Colonies derived from differentiated culture T6 are bigger than colonies from T6 SC. For B-C data are shown T6 for and HT29 and are representative of 2 independent and 1 single experiment for T6 and HT29, respectively. The number of spheres analysed for size is indicated on the Fig. Data is presented as mean ± SD, *P<0.05, **P<0.001, ***P<0.0001. (TIF) [file pone.0146052.s003.tif]

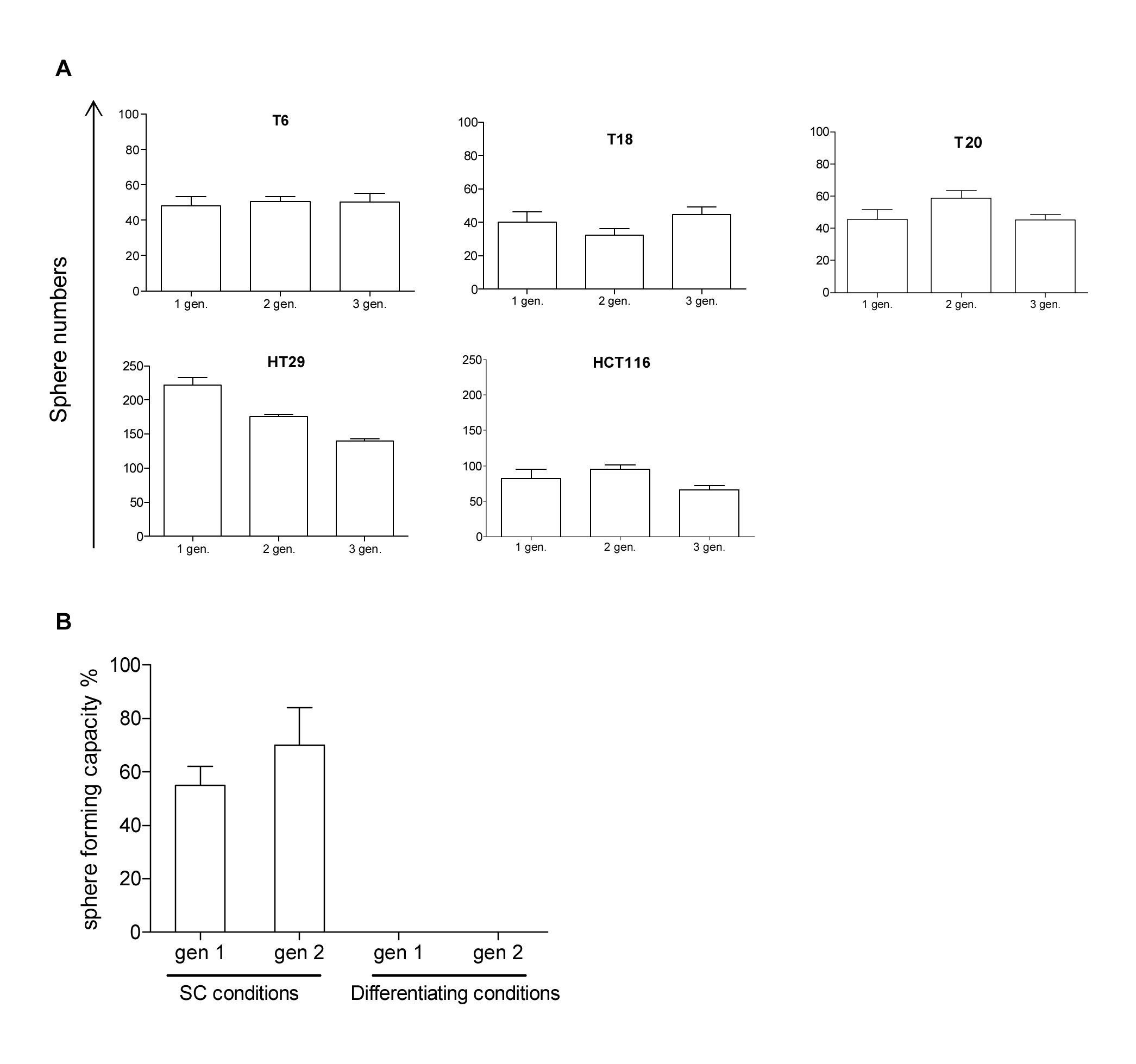

Supplement: S4 Fig — (A) High numbers of cells form spheres just by fusion and aggregation, and not due to increased self-renewal properties. Self-renewal capacity determined by the 1000 cell sphere formation assay of early passage SC grown from primary tumor tissue and CRC cell lines. Number of spheres observed by plating 1000 cells per well does not correlate to the results of the single cell assay presented in Fig 3, which might suggest that fusion and aggregation, rather than self-renewing capacity lead to sphere formation in the 1000 cell assay. Sphere formation was observed over several generations (gen.). Data are presented as mean ± SD. (B) Self-renewal capacity determined by the single cell assay of the differentiated counterparts (late passages) reversed to spheroid culturing conditions or maintained in differentiating culturing conditions, respectively. Sphere formation was observed over two generations (gen.). Data is shown for T18 and presented as mean ± SD, *P<0.05, **P<0.001. (TIF) [file pone.0146052.s004.tif]

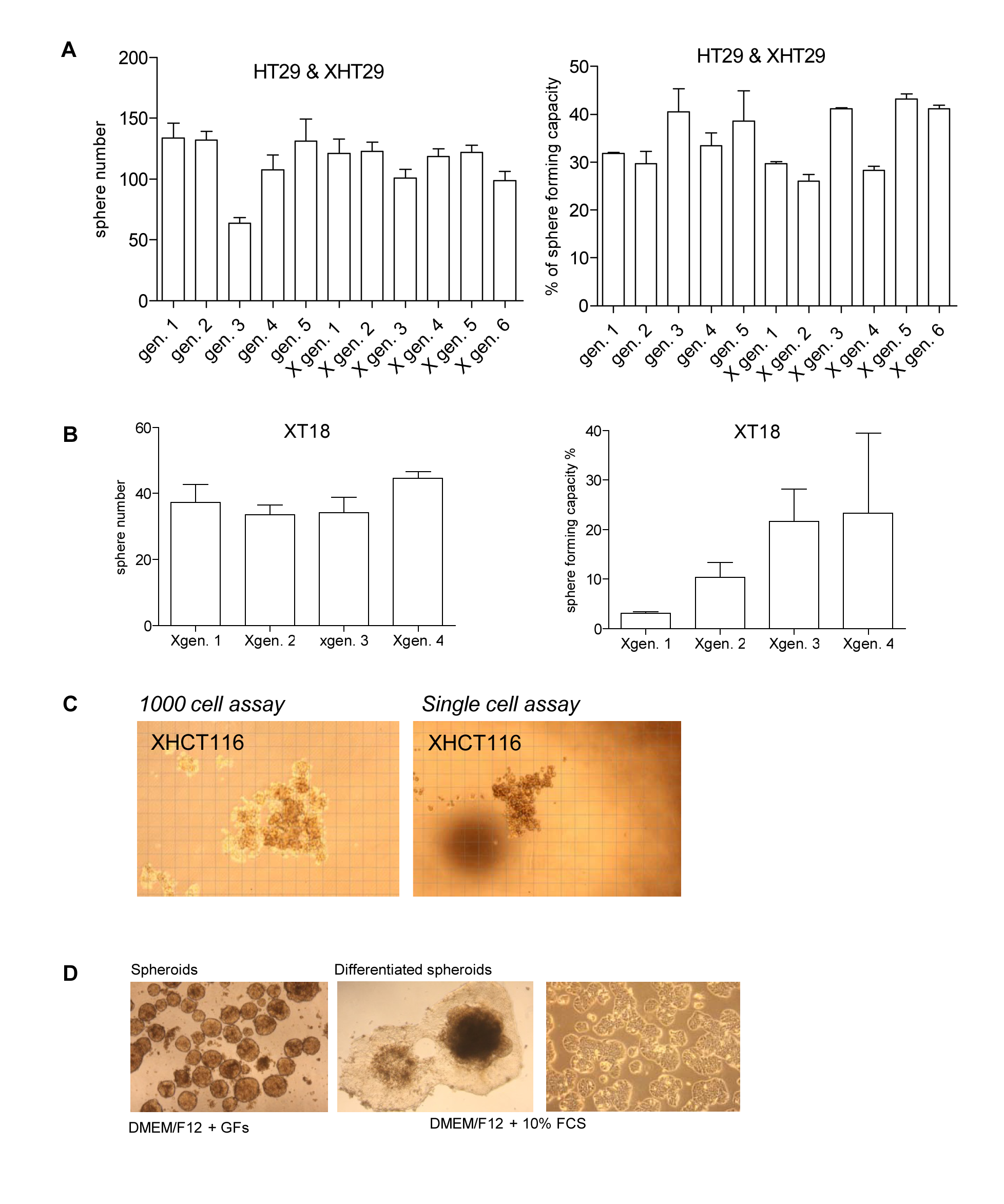

Supplement: S5 Fig — (A) and (B) Self-renewal capacity, shown by 1000 cell (left panel) and single cell (right panel) sphere forming assays, is not increased in xenograft-derived SC over several generations (gen.) compared to SC directly isolated from fresh tumor tissue. Data are presented as mean of 6 replicates ± SD. Xgen = xenograft-derived generation. (C) Xenograft-derived HCT116 SC grow as loosely packed aggregates that no longer resemble to spheres. (D) Morphological features of differentiated CRC SC. CRC spheroids adhere and differentiate when grown in medium supplemented with serum. (TIF) [file pone.0146052.s005.tif]

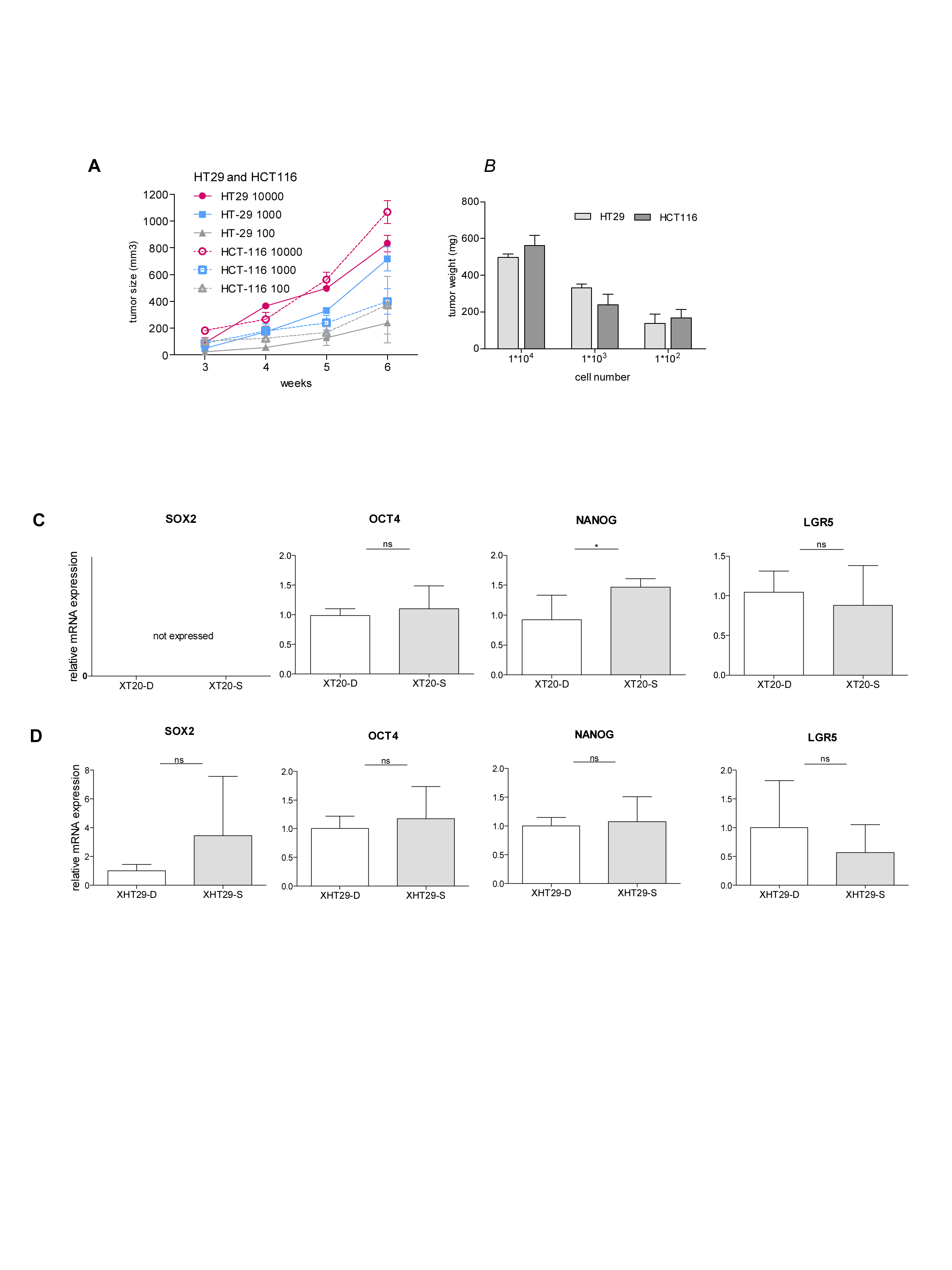

Supplement: S6 Fig — (A) SC derived from CRC cell lines are able to induce tumors in mice. (B) Tumor weight of the generated tumor xenografts. n = 5, data are presented as mean ± SEM. (C) and (D) SC-derived xenografts (S) and xenografts generated from their differentiated counterparts (D) display similar expression patterns of stemness genes SOX2, OCT4, NANOG and LGR5. Data are presented as mean ± SD, *P<0.05, ns = not significant, n = 5. (TIF) [file pone.0146052.s006.tif]

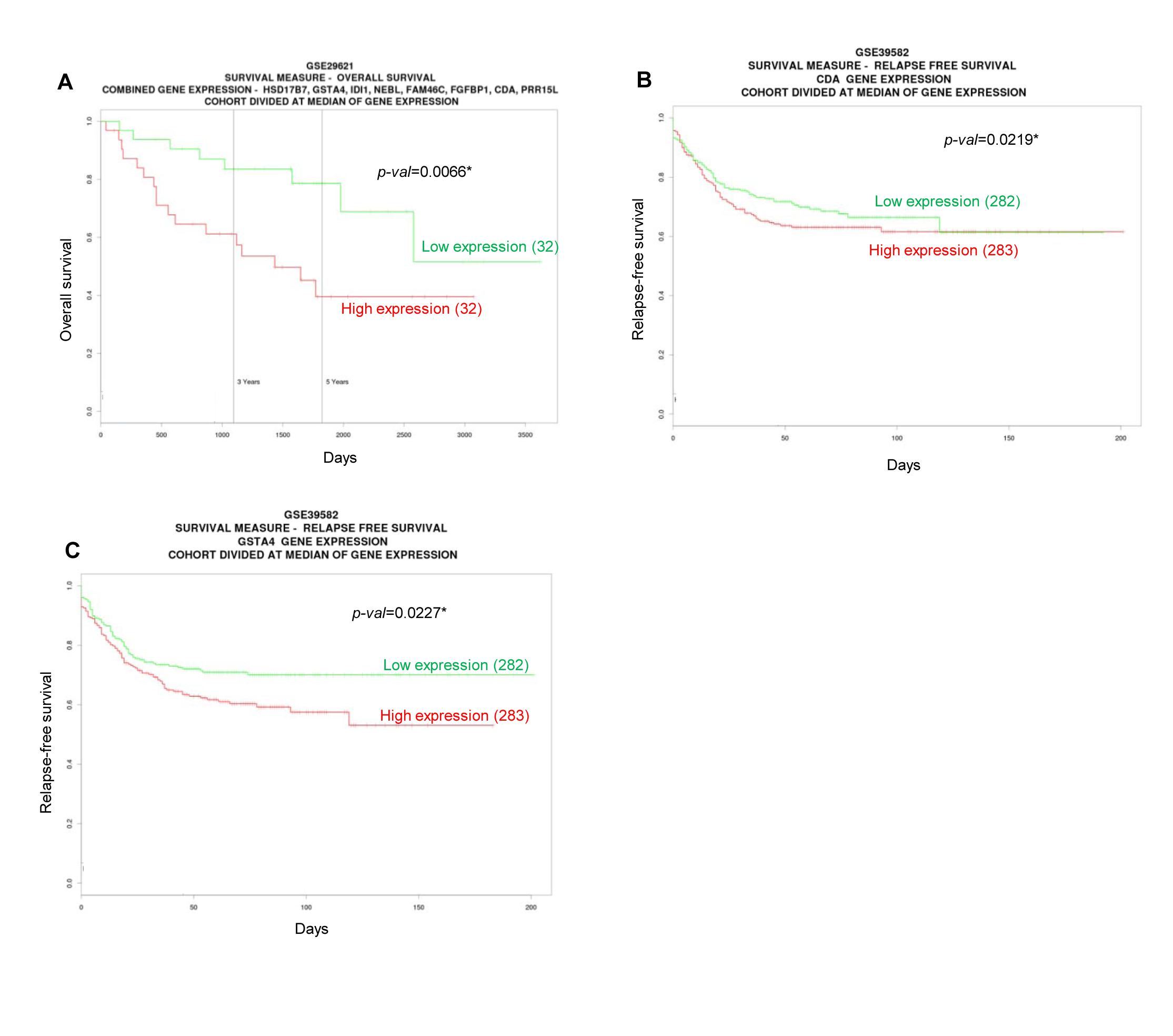

Supplement: S7 Fig — (A) Overall survival curves for CRC patients classified according to gene expression levels for the 8-gene sphere signature in the datasets GSE29621. (B-C) The expression of two genes from our identified gene signature (CDA & GSTA4) is linked to an increased risk of disease relapse in the dataset GSE39582. The number of patients in each group is mentioned within the brackets; significant p-value is indicated. SC = spheroid cultures, D = differentiated counterparts. (TIF) [file pone.0146052.s007.tif]
