# Supplementary material for: What Do We Learn from Spheroid Culture Systems? Insights from Tumorspheres Derived from Primary Colon Cancer Tissue
Source: PLoS One. 2016 Jan 8;11(1):e0146052. doi: 10.1371/journal.pone.0146052 (PMC4706382; doi:10.1371/journal.pone.0146052)
Supplement: S1 Table — (PDF) [file pone.0146052.s008.pdf]

S1A Table. Primary and secondary antibodies used for immunofluorescence analysis.	
Antibody	References
anti-SOX2 (1:50)	(Abcam, ab97959)
anti-OCT4 (1:50)	(Abcam, ab18976)
anti-LGR5 (1:50)	(Abcam, 75732)
anti-CK20 [Ks20.8] (1:200)	(Genetex, GTX72046)
Alexa fluor 594 goat anti-rabbit (1:500)	(Invitrogen, A11012)
Alexa fluor 488 goat anti-mouse (1:500)	(Invitrogen, A10667)

S1B Table. Primary antibodies and isotype controls used for FACS analysis.			
Antibody	References	Isotype control	References
CD133 (AC133) APC	Miltenyi Biotec, 130-090-826	Mouse IgG1-APC	Miltenyi Biotec, 130-090-214
CD44 PE-Cy7	BD Biosciences, 560533	PE-Cy™7 Mouse IgG2b, κ	BD Biosciences, 560542
CD24 FITC	BD Biosciences, 555427	FITC Mouse IgG2a, κ	BD Biosciences, 556652
CD24 PE	BD Biosciences, 555428	PE Mouse IgG2a, κ	BD Biosciences, 555574

S1C Table. Primer pairs used for RT-qPCR.		
Gene	Forward	Reverse
OCT4 (POU5F1)	5’-GAG-AAC-CGA-GTG-AGA-GGC-AAC-C-3’	5’-CAT-AGT-CGC-TGC-TTG-ATC-GCT-TG-3’
NANOG	5’-AAT-ACC-TCA-GCC-TCC-AGC-AGA-TG-3’	5’-TGC-GTC-ACA-CCA-TTG-CTA-TTC-TTC-3’
SOX2	5’-AGG-GGG-AAA-GTA-GTT-TGC-TGC-CT-3’	5’-TGC-CGC-CGC-CGA-TGA-TTG-TT-3’
LGR5	5’-AGC-CAT-GAC-CTT-GGC-CCT-GA-3’	5’-GCT-GTG-GAG-CCC-ATC-AAA-GCA-3’
EFF1A1	5’-TTG-TCG-TCA-TTG-GAC-ACG-TAG-3’	5’-TGC-CAC-CGC-ATT-TAT-AGA-TCA-G-3’
B-Actin	5’-GCA-AAG-ACC-TGT-ACG-CCA-ACA-3’	5’-ACA-CGG-AGT-ACT-TGC-GCT-CAG-3’
28S	5’-GAA-CTT-TGA-AGG-CCG-AAG-TG-3’	5’-ATC-TGA-ACC-CGA-CTC-CCT-TT-3’
YWHAZ	5’-ACT-TTT-GGT-ACA-TTG-TGG-CTT-CAA-3’	5’-CCG-CCA-GGA-CAA-ACC-AGT-AT-3’
CTNNB1 (β-catenin)	5’-GTG-CTA-TCT-GTC-TGC-TCT-AGT-A-3’	5’-CTT-CCT-GTT-TAG-TTG-CAG-CAT-C-3’
