# Supplementary material for: What Do We Learn from Spheroid Culture Systems? Insights from Tumorspheres Derived from Primary Colon Cancer Tissue
Source: PLoS One. 2016 Jan 8;11(1):e0146052. doi: 10.1371/journal.pone.0146052 (PMC4706382; doi:10.1371/journal.pone.0146052)
Supplement: S2 Table — (PDF) [file pone.0146052.s009.pdf]

S2 Table. Patient Characteristics.

Patients	Age/sex	Tumor site	TNM Stages		Tumor differentiation	Liver metastasis	MSS/MSI status
T6	84/m	left descending colon	Stage III C	T4aN2a	moderate	no	MSS
T18	82/m	right ascending colon	Stage II A	T3N0	well	no	MSS
T20	79/m	transverse colon	Stage IV A	T3N2bM1a	moderate	yes	MSS
T29	70/f	colon left angle	Stage II A	T3N0	well	no	MSS
T35	80/m	rectum	Stage III B	T3N1b	well	no	MSS

T*: Primary Tumor,* ***N****: Regional Lymph Nodes,* ***M****: Distant Metastasis,* ***MSS****: Microsatellite stability*
