# Supplementary material for: What Do We Learn from Spheroid Culture Systems? Insights from Tumorspheres Derived from Primary Colon Cancer Tissue
Source: PLoS One. 2016 Jan 8;11(1):e0146052. doi: 10.1371/journal.pone.0146052 (PMC4706382; doi:10.1371/journal.pone.0146052)
Supplement: S3 Table — (PDF) [file pone.0146052.s010.pdf]

S3 Table. *In vitro* limiting dilution assays of CRC spheroid cultures - Sphere forming cell (SFC) frequency.

	P1			P2			P3		
Colon cancer sample	SFC frequency 1 in x			SFC frequency 1 in x			SFC frequency 1 in x		
	Lower	**Estimate**	Upper	Lower	**Estimate**	Upper	Lower	**Estimate**	Upper
T6	12.19	**9.14**	6.85	11.46	**7.51**	4.92	20.10	**11.66**	6.77
T18	13.09	**8.47**	5.48	7.15	**4.54**	2.88	6.32	**4.45**	3.13
T20	1.78	**1.57**	1.40	3.15	**2.03**	1.43	2.09	**1.75**	1.49
HT29	3.21	**2.54**	2.01	2.68	**2.13**	1.69	1.58	**1.28**	1.03
HCT116	2.76	**2.26**	1.85	3.49	**2.69**	2.08	12.0	**7.9**	5.19
