# Supplementary material for: What Do We Learn from Spheroid Culture Systems? Insights from Tumorspheres Derived from Primary Colon Cancer Tissue
Source: PLoS One. 2016 Jan 8;11(1):e0146052. doi: 10.1371/journal.pone.0146052 (PMC4706382; doi:10.1371/journal.pone.0146052)
Supplement: S4 Table — (PDF) [file pone.0146052.s011.pdf]

**S4 Table.** ***In vivo*** **limiting dilution assays of CRC spheroid cultures – number of injected cells from dissociated spheroids and tumor formation occurrence in mice.**							***In vivo*** **limiting dilution assays of CRC spheroid cultures – cancer initiating cell (C-IC) frequency**		
	Cell number injected into NOD/SCID mice						C-IC frequency 1 in x		
CRC sample	**$1*10^4$**	**$2.5*10^3$**	**$1*10^3$**	**$1*10^2$**	**50**	**10**	Lower	**Estimate**	Upper
T6	5/5	-	6/6	6/6	5/6	6/9	33.4	**16.3**	8.10
T18	-	-	6/6	6/6	-	2/6	62.6	**22.1**	8.05
T20			5/5	6/6		5/6	15.7	6.1	2.60
HT29	2/2	3/3	4/4	3/3	-	-	214	1	1
HCT-116	4/4	-	4/6	3/4	-	-	1311	520	206
