# Supplementary material for: What Do We Learn from Spheroid Culture Systems? Insights from Tumorspheres Derived from Primary Colon Cancer Tissue
Source: PLoS One. 2016 Jan 8;11(1):e0146052. doi: 10.1371/journal.pone.0146052 (PMC4706382; doi:10.1371/journal.pone.0146052)
Supplement: S5 Table — A-D. Statistics of the sphere formation and 5-FU chemosensitivity assay. 2way ANOVA with Bonferroni post-tests, n = 8, *P<0.05, **P<0.01, ***P<0.001, S = Spheres, D = Differentiated counterpart. E-F Percentages of colony formation in SC and differentiated counterparts after 5FU treatment. (PDF) [file pone.0146052.s012.pdf]

S5A Table.

T6										
Days	ctrl S vs 7.5 μM S	ctrl S vs 15 μM S	ctrl S vs 25 μM S	ctrl S vs 50 μM S	ctrl S vs 80 μM S	ctrl D vs 7.5 μM D	ctrl D vs 15 μM D	ctrl D vs 25 μM D	ctrl D vs 50 μM D	ctrl D vs 80 μM D
1	**	*	ns	ns	ns	ns	ns	ns	ns	ns
2	ns	ns	ns	ns	ns	**	***	***	***	***
3	ns	ns	ns	ns	ns	*	***	***	***	***
4	ns	ns	ns	ns	ns	***	***	***	***	***
5	ns	ns	ns	ns	ns	**	***	***	***	***

S5B Table.

T18										
Days	ctrl S vs 7.5 μM S	ctrl S vs 15 μM S	ctrl S vs 25 μM S	ctrl S vs 50 μM S	ctrl S vs 80 μM S	ctrl D vs 7.5 μM D	ctrl D vs 15 μM D	ctrl D vs 25 μM D	ctrl D vs 50 μM D	ctrl D vs 80 μM D
1	ns	ns	ns	ns	ns	ns	ns	ns	ns	ns
2	ns	ns	ns	ns	ns	ns	ns	*	ns	ns
3	ns	ns	ns	ns	ns	***	***	***	***	***
4	ns	ns	ns	***	***	**	***	***	***	***
5	*	***	***	***	***	***	***	***	***	***

S5C Table.

T20										
Days	ctrl S vs 7.5 μM S	ctrl S vs 15 μM S	ctrl S vs 25 μM S	ctrl S vs 50 μM S	ctrl S vs 80 μM S	ctrl D vs 7.5 μM D	ctrl D vs 15 μM D	ctrl D vs 25 μM D	ctrl D vs 50 μM D	ctrl D vs 80 μM D
1	ns	ns	ns	ns	ns	ns	ns	ns	ns	ns
2	ns	ns	ns	ns	ns	ns	ns	***	***	***
3	ns	ns	ns	ns	***	*	***	***	***	***
4	ns	ns	**	***	***	***	***	***	***	***
5	ns	ns	**	***	***	***	***	***	***	***

S5D Table.

HT29										
Days	ctrl S vs 7.5 μM S	ctrl S vs 15 μM S	ctrl S vs 25 μM S	ctrl S vs 50 μM S	ctrl S vs 80 μM S	ctrl D vs 7.5 μM D	ctrl D vs 15 μM D	ctrl D vs 25 μM D	ctrl D vs 50 μM D	ctrl D vs 80 μM D
1	ns	ns	ns	ns	ns	ns	ns	ns	ns	ns
2	ns	ns	ns	ns	ns	ns	ns	ns	***	***
3	ns	ns	*	***	***	***	***	***	***	***
4	ns	*	***	***	***	***	***	***	***	***
5	ns	***	***	***	***	***	***	***	***	***

**S5E Table. Percentages of colony formation in T6 SC and differentiated counterpart**				
Cell doses	250	100	50	20
T6-D	55.2±1.13%	54±8.49%	64±11.31%	55±21.21%
T6-S	63.2±5.66%	73.5±3.54%	70±5.66%	65±14.14%
T6-D + 5FU	25.4±1.98%	48±5.66%	29±1.41%	55±7.07%
T6-S + 5FU	61.4±1.98%	60.5±13.44%	67±9.90%	45±14.14%

**S5F Table. Percentages of colony formation in HT29 SC and differentiated counterpart**				
Cell doses	250	100	50	20
HT29-D	53.4±2.55%	52±4.24%	51±12.73%	65±14.14%
HT29-S	61.2±3.39%	61.5±3.54%	77±7.07%	67.5±3.54%
HT29-D + 5FU	24.8±2.26%	29±1.41%	31±9.90%	37.5±3.54%
HT29-S + 5FU	51.4±3.11%	62±2.83%	71±9.90%	60±14.14%
