# Supplementary material for: What Do We Learn from Spheroid Culture Systems? Insights from Tumorspheres Derived from Primary Colon Cancer Tissue
Source: PLoS One. 2016 Jan 8;11(1):e0146052. doi: 10.1371/journal.pone.0146052 (PMC4706382; doi:10.1371/journal.pone.0146052)
Supplement: S6 Table — (PDF) [file pone.0146052.s013.pdf]

S6 Table. Genes from the 8-gene signature and their functions.			
Gene name	Known function	Cancer type	References
HSD17B7, NEBL	tumor growth	breast, AML	(1,2)
FAM46C	poor disease-free survival	myeloma	(3,4)
FGFBP1	tumor initiation, metastasis, poor prognosis	pancreas, colon, gallbladder, breast, liver	(5–8)
IDI1	chemoresistance	ovarian	(9)
CDA	tumor growth, chemoresistance	breast	(10–14)
GSTA4	chemoresistance	breast, ovarian	(15,16)
PRR15L	unknown	-	-
