# Supplementary material for: Correction: What Do We Learn from Spheroid Culture Systems? Insights from Tumorspheres Derived from Primary Colon Cancer Tissue
Source: PLoS One. 2016 Feb 29;11(2):e0150179. doi: 10.1371/journal.pone.0150179 (PMC4771198; doi:10.1371/journal.pone.0150179)
Supplement: S1 File — (PDF) [file pone.0150179.s001.pdf]

PLOS | ONE

RESEARCH ARTICLE

What Do We Learn from Spheroid Culture Systems? Insights from Tumorspheres Derived from Primary Colon Cancer Tissue

Komal Qureshi-Baig[1], **Pit Ullmann**[1], **Fabien Rodriguez**[1], **Sónia Frasquilho**[2], **Petr V. Nazarov**[3], **Serge Haan**[1], **Elisabeth Letellier**[1]*

1 Molecular Disease Mechanisms Group, Life Sciences Research Unit, University of Luxembourg, 6 Avenue du Swing, L-4367, Campus Belval, Luxembourg, **2** Integrated Biobank of Luxembourg, 6 rue Nicolas Ernest Barblé, L-1210, Luxembourg, Luxembourg, **3** Luxembourg Institute of Health, Genomics Research Unit, 84 Val Fleuri, L-1526, Luxembourg, Luxembourg

* elisabeth.letellier@uni.lu

CrossMark
click for updates

OPEN ACCESS

Citation: Qureshi-Baig K, Ullmann P, Rodriguez F, Frasquilho S, Nazarov PV, Haan S, et al. (2016) What Do We Learn from Spheroid Culture Systems? Insights from Tumorspheres Derived from Primary Colon Cancer Tissue. PLoS ONE 11(1): e0146052. doi:10.1371/journal.pone.0146052

Editor: Alessandro Datti, Lunenfeld-Tanenbaum Research Institute, CANADA

Received: August 11, 2015

Accepted: December 11, 2015

Published: January 8, 2016

Copyright: © 2016 Qureshi-Baig et al. This is an open access article distributed under the terms of the Creative Commons Attribution License, which permits unrestricted use, distribution, and reproduction in any medium, provided the original author and source are credited.

Data Availability Statement: All relevant data are within the paper and its Supporting Information files. All gene expression files are available from the ArrayExpress database under accession number E-MTAB-3575.

Funding: The authors would like to thank the Fondation Cancer (grant F1R-LSC-PAU-13HY2C) for funding this study and the Fonds National de la Recherche (FNR) for supporting KB and PU under the AFR grant scheme. We also thank the Integrated Biobank of Luxembourg (IBBL) for supporting this study. The authors are also grateful to the Fondation

Abstract

Due to their self-renewal and tumorigenic properties, tumor-initiating cells (TICs) have been hypothesized to be important targets for colorectal cancer (CRC). However the study of TICs is hampered by the fact that the identification and culturing of TICs is still a subject of extensive debate. Floating three-dimensional spheroid cultures (SC) that grow in serum-free medium supplemented with growth factors are supposed to be enriched in TICs. We generated SC from fresh clinical tumor specimens and compared them to SC isolated from CRC cell-lines as well as to adherent differentiated counterparts. Patient-derived SC display self-renewal capacity and can induce serial transplantable tumors in immuno-deficient mice, which phenotypically resemble the tumor of origin. In addition, the original tumor tissue and established SC retain several similar CRC-relevant mutations. Primary SC express key stemness proteins such as SOX2, OCT4, NANOG and LGR5 and importantly show increased chemoresistance ability compared to their adherent differentiated counterparts and to cell line-derived SC. Strikingly, cells derived from spheroid or adherent differentiating culture conditions displayed similar self-renewal capacity and equally formed tumors in immune-deficient mice, suggesting that self-renewal and tumor-initiation capacity of TICs is not restricted to phenotypically immature spheroid cells, which we describe to be highly plastic and able to reacquire stem-cell traits even after long differentiation processes. Finally, we identified two genes among a sphere gene expression signature that predict disease relapse in CRC patients. Here we propose that SC derived from fresh patient tumor tissue present interesting phenotypic features that may have clinical relevance for chemoresistance and disease relapse and therefore represent a valuable tool to test for new CRC-therapies that overcome drug resistance.

du Pélican de Mie et Pierre Hippert-Faber under the aegis of Fondation de Luxembourg for supporting KB and PU. The funders had no role in study design, data collection and analysis, decision to publish, or preparation of the manuscript.

Competing Interests: The authors have declared that no competing interests exist.

Abbreviations: 5-FU, 5-Fluorouracil; CRC, colorectal cancer; TIC, tumor-initiating cell; CSC, cancer stem cell; SC, spheroid cultures; SFC, sphere forming cell; TIC, tumor-initiating cell.

Introduction

Colorectal carcinoma (CRC) is the third most frequently diagnosed cancer type for both men and women and the second most common cause of cancer mortality in Western countries [1]. Despite great progress made during the last decades, a lot of controversy still remains over the backgrounds of cancer onset, metastasis and CRC progression. The two dominant concepts of carcinogenesis postulate stochastic (clonal evolution model) and hierarchic (cancer stem cell model) organization of tumors. According to the latter, subsets of cells, the so-called tumor-initiating cells (TICs) also known as cancer stem cells (CSCs) are responsible for tumor evolution [2].

TICs have first been described in the frame of hematopoietic malignancies [3]. A few years later, TICs were also identified in a wide array of solid tumors such as, breast [4,5], skin [6], brain [7–9], pancreas [10], lung [11] and colon [12,13]. TICs are defined by their (1) self-renewal, (2) differentiating and (3) tumor-initiating capacity. They have been described to propagate tumors that are capable of recapitulating the heterogeneity of primary tumors [3]. The high tumorigenic potential of TICs is aggravated by their strong resistance to radio- and chemo-therapy. TICs are able to evade DNA damage during radiation and chemotherapy by reduction of ROS and enhanced activity of DNA checkpoint kinases [14]. The remaining subset of TICs might induce the formation of new tumors, thereby leading to a rapid relapse of the malignancy [15]. As a result, TIC-specific treatments could potentially lead to a reduced risk of tumor recurrence and an improved prognosis for CRC patients. Interestingly, various recent studies support the clinical relevance of targeting TIC-associated genes [16].

Despite significant advances in colon TIC research, the isolation, identification and characterization of colon TICs remains incompletely established. Different strategies can be adopted to gain colon TICs out of tumor tissue. Isolation techniques for TICs are based either on their immunogenic or functional properties [17]. The antigenic approach takes advantage of a variety of cell surface markers, for example prominin-1 (commonly known as CD133), CD44, CD34, CD24, epithelial-specific antigen (EpCAM/ESA), CD166, CD29, Lgr5, CD49f and ALDH-1 [17]. Functional isolation of TICs relies on diverse characteristics, such as anchorage-independent growth, chemoresistance, self-renewal, asymmetric division, and pluripotency. Over the last years spheroid cultures (SC) that rely on the anchorage-independent growth properties of stem cells, have been used to enrich for TICs in brain [18,19], breast [5,20], and colon [12,21,22] tissue. It is well accepted that SC preserve more faithfully the characteristics of original tumors, including gene expression profiles, tumor heterogeneity and tumor morphology, compared to regular adherent cultures [12,19,21–25]. Additionally, spheroids mirror the 3D cellular context and relevant pathophysiological gradients of *in vivo* tumors [26]. However, to which extent sphere formation assays favor the enrichment of TICs is not fully clear yet. First, it is noteworthy that spheroids also contain differentiated tumor cells [21,22,27,28]. Second, some studies on CRC could indeed demonstrate that SC possess TIC characteristics [12,21,29–31], whereas others could not find any enrichment when comparing them to adherent differentiated cultures [31–36]. Importantly most of the aforementioned studies are based on cell lines. It can be speculated that the inconsistent observations gained with cell lines might be due to phenotypic differences between selected clones that might have occurred over long periods of cell culture [37].

Considering these conflicting results, we decided to characterize SC from different origins in the light of TIC enrichment. In contrast to traditional cell lines, patient-derived primary cultures reflect the heterogeneous nature of tumor biology, as it exists in patients. Thus, the importance of this study is to characterize generated SC directly derived from fresh surgical specimens and compare them to their adherent differentiated counterpart as well as to SC

derived from CRC cell lines. We want to determine if SC exhibit features of stemness, including self-renewal, stem cell marker expression, differentiation potential, resistance to chemotherapy and tumorigenicity *in vivo*.

Material and Methods

Generation of primary SC and their differentiated counterparts

Human colon tissue samples were collected by the Integrated Biobank of Luxembourg (IBBL, www.ibbl.lu) in accordance with institutional guidelines. All human samples used in the scope of this work were donated freely and written informed consent was obtained from the donor for the use of this sample in research. Ethical approval was obtained from the Comité National d'Ethique de Recherche, Luxembourg (Reference 201009/09). Freshly resected colon tumor tissue from 35 CRC patients was collected and immediately processed in culture. Tissue was washed several times in serum-free Dulbecco's modified Eagle medium (DMEM)-F12 supplemented with antibiotic-antimycotic agent (Invitrogen). The specimens were minced into 1–2 mm^3 pieces followed by incubation in collagenase type IV (0.05 mg/ml; Invitrogen) and hyaluronidase (2 μg/mL; Sigma) for 1 hour at 37°C. Single cell suspension was obtained by mixing every 15 minutes and by filtration through a 70 μm cell strainer (BD Biosciences). Primary colon SC were maintained in ultra-low attachment (ULA) cell culture vessels (Corning) in serum-free stem cell medium containing DMEM-F12 supplemented with B-27 (1x) and N-2 (1x) supplements (Invitrogen), BSA (4 mg/mL; Roth), non-essential amino acids (1x; Sigma), glucose 0.15% (Sigma), Insulin (4 U/L; Sigma), Heparin (4 μg/mL), N-Acetylcystein (1 mM; Sigma), EGF (20 ng/mL; Biomol), bFGF (20 ng/mL; Miltenyi Biotec), and penicillin/streptomycin (1x; Lonza). This medium will further be referred to as stem cell medium (SCM). For characterization purposes, we only used early passage SC within this study (not more than 15 passages).

We generated adherent-growing differentiated cultures from SC at very early passages. For this, differentiating conditions were applied: early spheroids were dissociated and cultured in DMEM-F12 supplemented with 10% FCS and 1% penicillin/streptomycin in regular cell culture vessels. In contrast to SC, differentiated cultures grow as adherent cells. These cultures were maintained under differentiating conditions for at least 5 passages before being used for experiments.

Spheroid cultures derived from CRC cell lines

HT29, HCT116, LS174t, SW480, and SW620 CRC cell lines were obtained either from the American Type Culture Collection (ATCC, Rockville, USA) or the German Collection of Microorganisms and Cell Cultures (DSMZ, Braunschweig, Germany) and maintained in recommended culture conditions. For the culture of SC, cell lines were grown in serum-free DMEM-F12 supplemented with B-27 (1x; Invitrogen), Insulin (4 U/L; Sigma), Heparin (4 μg/ml; Sigma), EGF (20 ng/mL; Biomol), bFGF (20 ng/mL; Miltenyi Biotec), and penicillin/streptomycin (1x; Lonza). Cell line identity was confirmed by short-tandem repeat genotyping at DSMZ.

3D spheroid invasion assay

5000 cells of SC were plated in round-bottom 96-well ULA plates in 100 μl DMEM-F12 medium with 1% penicillin/streptomycin. After 3 days of sphere formation, 10% FCS and 1.25 mg/ml collagen (PureCol, CellSystems) were added. After 1h of solidification, the spheroid/collagen suspension was overlaid with 100μL/well DMEM-F12 with 10% FCS. Invasion was monitored by measuring the maximal spheroid outgrowth diameter after 7 days.

Cell growth and proliferation

Adherent cells were seeded in 6-well plates and SC in ULA 6-well plates in their respective medium. After 5 days, cells were dissociated into single-cell suspension, stained with Trypan blue and counted with a cell counting device (CEDEX, Roche).

In vitro limiting dilution sphere formation assays

SC were dissociated in TrypLE Express (Gibco) by pipetting up and down for 1 minute, incubated at 37°C for 3 minutes and passed through a 40 μm cell strainer (BD Biosciences) to obtain single cell suspensions. ***Single cell assay***: Cells were seeded at a density of 1 cell per well in a 96-well plate at a final volume of 100 μl. Single cell seeding efficiency was approximately 30% and only wells that initially contained single cells were evaluated for subsequent analyses. Spheroids were counted after 10–14 days, depending on the growth rate of the different primary SC. 50–60 single cells per condition were analyzed for sphere formation and only spheres bigger than 50 μm in size were included in the analyses. The extreme limiting dilution analysis ELDA software (http://bioinf.wehi.edu.au/software/elda/) [38] was used to determine the estimated stem-cell frequency for single cell assays. ***1000 cell assay***: 1000 cells/well were seeded in an ULA 6-well plate and spheroids were counted after 7 days.

Immunofluorescence

Immunofluorescence was performed on cytospins (EZ Cytofunnels) (Thermo Scientific) for SC and on coverslips for adherent cell cultures. Samples were fixed with ice-cold methanol for 10 minutes at -20°C, blocked with a 3% BSA/PBS solution followed by incubation overnight at 4°C with the primary antibody. Secondary antibody was applied for 1 hour at room temperature; samples were mounted with DAPI-containing Fluoromount G (Southern Biotech) and analyzed on a confocal microscope (Zeiss LSM 510 Meta). To certify that the staining was positive throughout the whole spheroid, z-stacks were performed. The antibodies used are listed in S1A Table.

Fluorescence activated cell sorting and flow cytometry

For flow cytometry, samples were prepared as previously described (Shmelkov et al, 2008) and run on a FACS Canto II. The primary antibodies used are listed in S1B Table.

Real-time qPCR

AllPrep extraction kit (Qiagen, Hilden, Germany) was used to extract RNA. cDNA was obtained by reverse transcription using the high capacity cDNA reverse transcription kit (Applied Biosystems). ABsolute Blue qPCR SYBR Green Low ROX Mix (Thermo Scientific), 2.5 pmol of each primer and 5 ng of cDNA per reaction was used and the reaction was run on a 7500 FAST Real time PCR Detection System (Applied Biosystems) cycler with the following settings: 40x (95°C for 30 sec, 60°C for 30 sec and 72°C for 30 sec). Expression levels of the gene of interest were normalized against multiple reference genes [39] using the qbase+ software [40], according to the MIQE guidelines. The used reference genes were: EFF1A1, B-Actin, 28S, YWHAZ. Primer pairs used for RT-qPCR are shown in S1C Table.

Colony formation assay

Cells derived from SC or differentiated cultures were seeded at different densities of 250, 100, 50, 20 cells/well in differentiating medium containing serum. After 10 days, colonies were stained and counted under a microscope.

Chemosensitivity assays

In order to assess chemoresistance we applied different assays. The first assay was based on a 3D spheroid system, where cells derived from SC or differentiated counterparts were seeded in a round-bottom 96-well ULA plate at a density of 5000 cells/well in serum-free DMEM-F12 supplemented with 1% penicillin/streptomycin. After 3 days of spheroid formation, 5-Fluorouracil (5-FU) (Sigma) was added at varying concentrations. Sphere size was measured every 24 hours for 5 days and sphere shrinkage was assessed by determining the relative sphere size of control and treated conditions. The second assay was based on a 2D monolayer system using the cell proliferation reagent WST-1 (Roche, Germany). For this, 30.000 cells of both SC and the differentiated counterparts were plated in 96-well plates in 200 μL of DMEM-F12 medium, supplemented with 10% FCS and 1% penicillin/streptomycin medium with or without 50 μM 5-FU. WST-1 was added at a 1:10 final concentration after 5 days and incubated for 1 h at 37°C and the relative survival was determined. In addition we performed single cell and colony forming assays in presence of 5μM 5-FU for the assessment of chemoresistance.

In vivo tumor formation assays

Non-obese diabetic/severe-combined immunodeficient (NOD/SCID) mice were obtained from Harlan Laboratories Netherlands and experiments performed according to all applicable laws and regulations subsequent to approval by the institution's animal care and ethical committee of the University of Luxembourg (Permit Number: 14-MDM-02). Tumor-initiating capacity of SC in NOD/SCID mice was assessed by preparing serial dilutions of cells (10. 000, 1000, 100 and 10 cells; 5–6 injections/cell dose). Single cells were resuspended in 100 μL of 1:1 mixed serum-free medium and matrigel (BD Biosciences) and injected subcutaneously in the flank of 6-week-old mice. Tumor growth was followed 1–2 times a week and tumor volume was calculated by the formula $L^*W^2/2$. Any mice showing severe signs of weight loss or distress or a tumor size reaching 1000 mm^3 were removed from the study and euthanized to avoid unnecessary pain and discomfort according to the ethical guidelines. For serial transplantations, tumors were explanted, dissociated in culture and re-injected in second recipient mice. Sections of generated xenografts were stained with eosin and hematoxylin and analyzed by a pathologist. To directly compare SC with their differentiated counterpart, we chose the lowest dose tested (10 cells/injection for T20 and 100 cells/injection for HT29; n = 5) and cells derived from differentiated or spheroid cultures were injected on right and left flank respectively.

Microarrays and mutation profiling

The microarrays were performed at the Luxembourg Institute of Health (LIH) after RNA quality and purity check using a 2100 Bioanalyzer (Agilent Technologies). Affymetrix gene chip Human Gene ST v2.0 arrays were prepared in accordance with the standard protocol. Microarray data were preprocessed using the Robust Multiarray Analysis (RMA) algorithm with GC-correction using commercial software Partek Genomic Suite (version 6.6, Copyright 2015, Partek Inc., St. Louis, MO, USA). Three replicates were run for all conditions for microarrays except T18 for which we ran 4 technical replicates. We had to withdraw one sample of the HCT116 cell line, because of its low quality after hybridization. Data were analyzed and the results visualized in R/Bioconductor (version 3.1.2). Microarray data are available in the ArrayExpress database under accession number E-MTAB-3575. Top mutations were assessed using the TruSeq Amplicon-Cancer panel (TSACP) with the MiSeq platform according to the Illumina guidelines with the following criteria: depth of > 1000 and a variant frequency of > 0.05. For ethical purposes we were not able to include the mutational profile for patient T6.

Differential gene expression and survival analysis

Microarrays (ArrayExpress database, accession number E-MTAB-3575) were performed in accordance with standard protocols (see supplementary material and methods section). Differentially expressed genes between SC and the respective differentiated counterparts were determined using linear modelling with empirical Bayesian approach realized in *limma* package (http://www.bioconductor.org/packages/ release/bioc/html/limma.html). A common sphere gene signature between primary and cell line derived SC was determined as intersection of up-regulated genes with FDR<0.05 and log-fold change log2FC>0.6. Two independent publicly available datasets from a collection available in the database PROGgeneV2 [41] containing data on the overall survival of CRC patients were used to generate survival curves: GSE17536 [42] composed of 177 patient samples and GSE29621 [43] consisting of 64 patient samples. We found a significant association with poor patient survival in these 2 datasets among a collection available in the aforementioned tool. The dataset GSE39582 [44] consisting of 566 patient samples was used to investigate the effect of the identified sphere gene signature on the disease-free survival.

Statistical analysis

GraphPad Prism 5 software was used for statistical analysis. We used unpaired Student t test to compare 2 conditions and 2-way ANOVA test with Bonferroni post-tests to compare treatment effects over time. All experiments were performed in at least 3 independent experiments unless otherwise stated.

Results

Generated SC derived from primary tumor tissue retain characteristics of original tumors

We first assessed the ability to enrich for TICs in CRC specimens using several of the previously reported potential surface markers. The expression of putative stem cell markers CD44, CD24, EpCAM, CD166 and CD133 was analyzed by flow cytometry. We found that patient biopsies are highly heterogeneous in terms of phenotypic markers (S1 Fig), questioning their reliability for the identification of TICs. We further decided to sort cells directly from patient- or cell line-derived SC according to their CD133 expression and performed functional single cell assays. The sphere forming cell (SFC) frequency was similar among the CD133 low, medium and high population (S1 Fig), suggesting that CD133 expression does not correlate to enhanced sphere-forming capacity. Along the same line, several *in vivo* studies show that CD133+ and CD133- cells form tumors with similar efficiency [34,45,46]. Overall, these results suggest that putative TIC markers are not reliable for the identification of TICs in our system.

SC may reflect tumor heterogeneity as it exists in patients and have been described to be enriched in TICs. We collected fresh colon cancer tissue from 35 patients who had not received chemotherapy or radiotherapy prior to surgery. After enzymatic digestion, single cell suspension was plated in SCM in order to promote spheroid growth. Tumor specimens of 5 out of 35 patients (15% efficiency) led to stable primary SC that we were able to maintain in culture for extended passages (more than 20 passages) (Fig 1*A* and S2 Table). The remaining colon cancer tissues did not lead to sphere formation or lost their sphere-forming capacity after a few passages. It is noteworthy that the success rate observed is comparable to other attempts to establish SC from colorectal tumor tissue [47,48]. The biopsies that gave rise to stable SC were of various histological stages of CRC (S2 Table). Three primary SC T6, T18 and T20, which represent stage IIIC, stage IIA and stage IVA CRC respectively, were chosen for characterization (S2

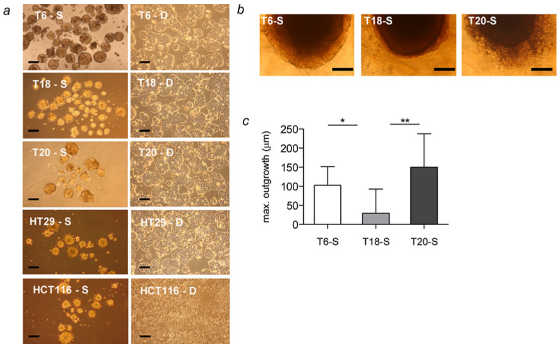

Fig 1. Description of generated SC. (*a*) Morphological features of 2 CRC cell lines (HT29, HCT116) and 3 primary patient tumors (T6, T18, T20) grown as SC (S, left panel) and their differentiated counterparts (*D*, right panel). (*b*) 3D spheroid invasion assay of SC derived from primary tumor tissue T6, T18 and T20. Representative pictures of embedded spheroids after 7 days of invasion. (*c*) Quantification of the maximal spheroid outgrowth diameter (µm) after 7 days of invasion. Representative figure of 3 independent experiments, scale bar = 100 µm. Data are presented as mean ± SD (n = 8); *P<0.05, **P<0.01.

doi:10.1371/journal.pone.0146052.g001

Table). Interestingly, SC derived from T6 and T20 patients showed an increased invasive capacity compared to the T18 spheroid culture, which was derived from an earlier stage, namely stage II (Fig 1*B* and 1*C*). This result suggests that SC derived from primary tumors may retain the invasive properties of their tumor of origin. However, this observation needs to be further addressed in much larger cohort studies. Notably, several CRC-relevant mutations detectable in the tumor of origin were also present in the primary SC (S2 Fig). We additionally established adherent differentiated counterparts of the primary SC (Fig 1*A* and Material and Methods section). The comparison of SC with their respective differentiated counterparts from the same patient might allow studying SC-specific properties in regard to TICs. In presence of serum, SC change their phenotype and grow as an adherent cell layer. Interestingly, adherent differentiated counterparts showed increased proliferation compared to SC (S3 Fig). Besides using primary tumor tissue, we also tested the ability of CRC cell lines to form SC. HT29, HCT116, LS174t, SW480 and SW620 cell lines gave rise to spheres over several passages while maintained in SCM (Fig 1A and data not shown). Consistent to SC derived from primary tissue, HT29- and HCT116- derived SC displayed a lower proliferation rate compared to their parental adherent counterpart (S3 Fig). This observation, which is in line with previous studies [35,36], reveals that upon differentiation, TICs acquire increased proliferative properties. In the following experiments, we focused on the extensive characterization of 3 SC established from primary patient material and 2 SC derived from CRC cell lines, namely HT29 and HCT116.

Spheroid cultures show expression of stemness proteins

In order to investigate in more detail the phenotypic differences between SC and the respective differentiated counterparts, we analyzed the expression of key stemness proteins such as SOX2, OCT4, NANOG and LGR5 as well as CK20, a key epithelial differentiation marker. The transcription factors OCT4, SOX2 and NANOG are known as master regulators of pluripotency and are responsible for maintaining an undifferentiated state [49] as well as favoring self-renewal capacity of TICs [50]. SC displayed high levels of stemness proteins whereas CK20 was barely expressed (Fig 2*A*). Importantly, the adherent differentiated counterparts expressed CK20 and lost the expression of key stemness proteins. Similarly to protein levels, the expression of key stemness genes was increased in SC compared to differentiated cultures (Fig 2*B*).

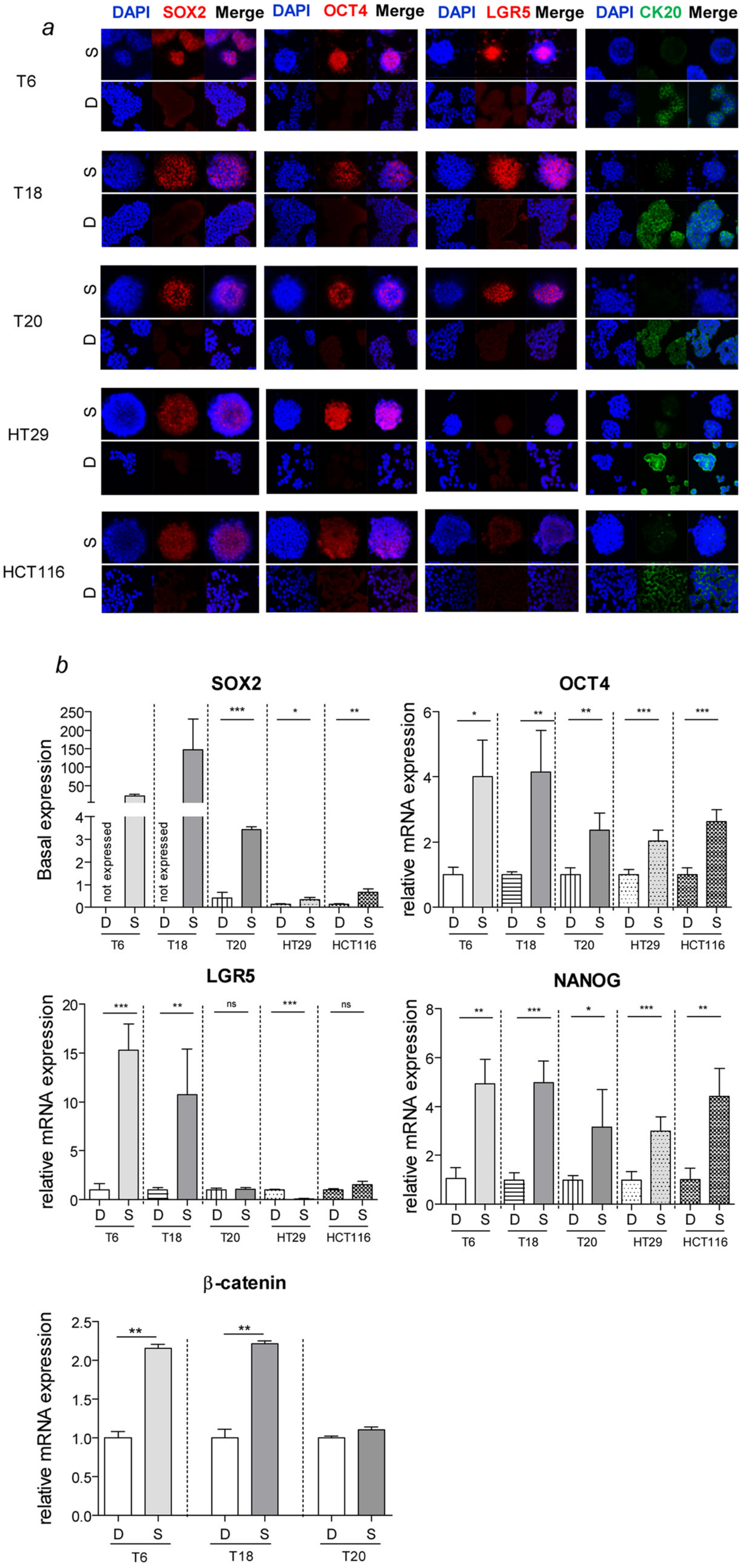
a
DAPI SOX2 Merge DAPI OCT4 Merge DAPI LGR5 Merge DAPI CK20 Merge
T6
T18
T20
HT29
HCT116
S
D
b
SOX2
Basal expression
not expressed
OCT4
LGR5
NANOG
β-catenin
relative mRNA expression
D S
T6 T18 T20 HT29 HCT116
ns

Fig 2. Increased expression of stemness regulators in SC. (*a*) Immunofluorescence staining of SC reveals an increased expression of key stemness proteins SOX2, OCT4 and LGR5, and a decreased expression of the CRC differentiation marker CK20 compared to adherent differentiated counterparts. NTERA-2 human embryonal carcinoma cells were used as positive control for staining and showed similar signal intensity for all assessed pluripotency markers (data not shown). Magnification 40x. (*b*) Gene expression of key stemness genes of SC and differentiated counterparts. Representative figure of 3 independent experiments. Data are presented as mean ± SD, *P<0.05, **P<0.01, ***P<0.001, ns = not significant.

doi:10.1371/journal.pone.0146052.g002

Interestingly, *LGR5* is highly expressed in primary SC, in contrast to SC derived from CRC cell lines (Fig *2A and 2B*). We also assessed the expression of β-catenin, a functional marker of CRC stemness, and found a higher expression in SC (Fig *2B*). Altogether, these data show that SC display higher levels of stemness and lower levels of epithelial differentiation markers.

Spheroid cultures and adherent differentiated counterparts display similar self-renewal and tumor-initiation capacity

We were interested in determining functional differences in regard to TIC properties between SC and adherent differentiated counterparts. At first, we tried to perform sphere formation assays by plating different cell densities. Many studies on TICs show self-renewal capacity using sphere formation assays by seeding high numbers of cells per well. However, in our experience and as already mentioned by others [33], plating high cell densities leads to sphere formation through aggregation and fusion followed by subsequent proliferation rather than through self-renewal capacity of a cell, thereby falsifying the sphere forming capacity results (S4A Fig). Thus, to ensure true clonality and not fusion or aggregation of cells we performed sphere formation assays at the single cell level. The 3 stable primary SC and the 2 SC derived from CRC cell lines all displayed self-renewing capacity that was maintained over several passages (Fig *3A*). For patient T6, there was 1 sphere forming cell (SFC) in 9.14 cells for passage 1, 1 SFC in 7.51 cells for passage 2, and 1 SFC in 11.66 cells for passage 3 (S3 Table). Strikingly, T20, derived from a patient who had already metastasis at the time of resection, showed an increased SFC frequency compared to T6 and T18 (S3 Table). Interestingly, the SFC frequency

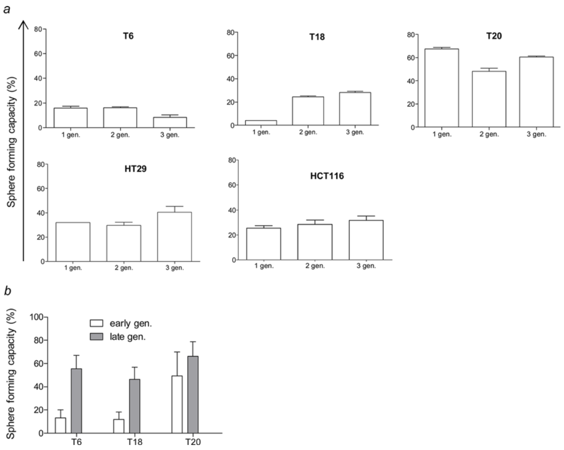

Fig 3. Self-renewal capacity is maintained over several passages. (*a*) Sphere formation rates were determined over several generations (gen.) by seeding single cells of early passage SC grown from primary tumor tissue and CRC cell lines. Data are presented as mean ± SD. (*b*) Self-renewal capacity of early (passage 5) and late (passage 15–25 depending on the tumor) SC from primary tumor tissue. Data are presented as mean with 95% confidence interval.

doi:10.1371/journal.pone.0146052.g003

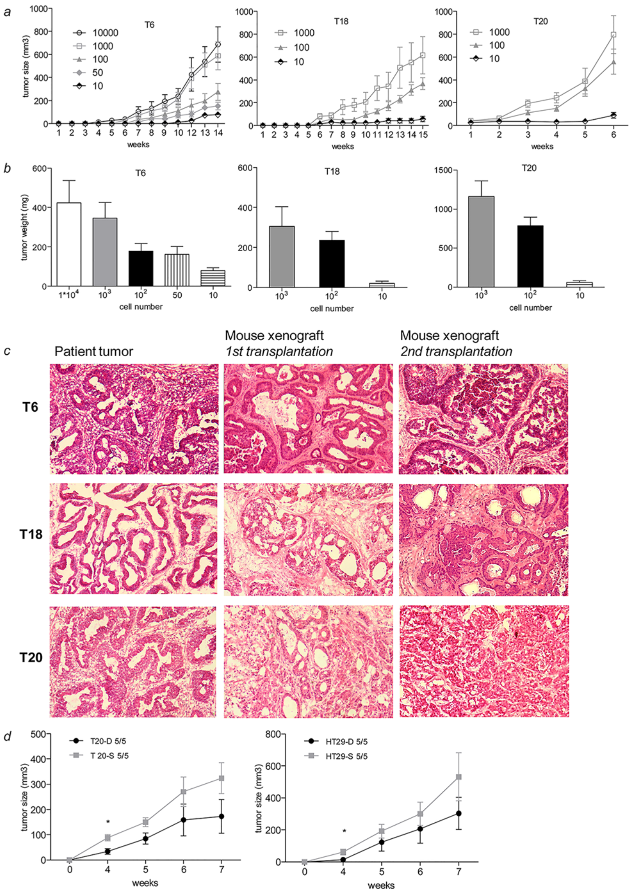

Fig 4. SC are highly tumorigenic but do not show differences in tumor incidence compared to adherent differentiated counterparts. (*a*) Primary SC induce tumors in mice. (*b*) Tumor weight of the generated tumor xenografts from primary SC. (*c*) Xenograft derived from primary SC recapitulate the phenotype of the primary tumor after serial transplantation in mice. Magnification 10x. (*d*) Tumor growth in mice after injection of SC and their differentiated counterpart. Tumor incidence is indicated within the graph. Data are presented as mean ± SEM, *P<0.05.

doi:10.1371/journal.pone.0146052.g004

rises from early passages (passage 5) to late passages (passage 15–25), thus suggesting that cultures maintained in stem-cell enriching conditions show increased TIC behavior over time (Fig 3B). Even after long-term culture in SCM, SC that are transferred to differentiating culture conditions have still the capacity to adhere and morphologically resemble the differentiated counterpart or the parental cell line (Fig 1A and S5D Fig).

Next, we studied the tumorigenic potential of SC. Primary SC generated from tumor tissue of patients T6, T18 and T20 were able to induce tumor formation in NOD/SCID mice with cell numbers ranging from 10.000 cells to 10 cells per injection (Fig 4A). TIC frequency varied from 1 in 6 to 1 in 22 spheroid cells depending on the respective patient sample (S4 Table). Similarly, SC derived from CRC cell lines also initiated tumor growth in mice with cell numbers ranging from 10.000 cells to 100 cells (S6A Fig and S4 Table), though HCT-116 SC had a lower tumor incidence compared to primary SC (S4 Table). In both cases, tumor weight nicely correlated with injected cell numbers (Fig 4B and S6B Fig). Interestingly, primary SC established from tumor specimen of patient T20 were able to induce tumor formation in mice much faster with higher tumor incidence compared to T6 and T18 SC (Fig 4A and S4 Table). The phenotype of T20-derived SC, which is characterized by high SFC frequency, tumor incidence

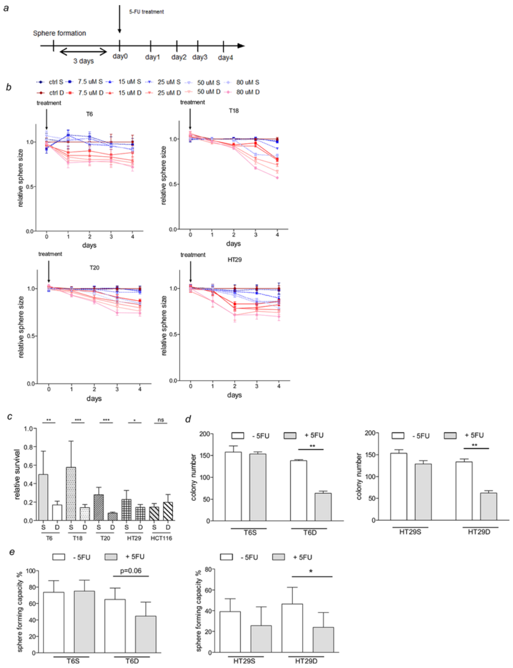

Fig 5. SC are more resistant to 5-FU treatment. (*a*) Experimental layout of the sphere formation and chemosensitivity assay. (*b*) Relative sphere size of SC and their differentiated counterparts exposed to different concentrations of 5-FU over 5 days. Representative figure of 2 independent experiments. Statistics are shown in S5A–S5D Table. (*c*) Relative survival of SC and their differentiated counterparts after exposure to 50 μM 5-FU for 5 days. Representative figure of 2 independent experiments is shown. Data are presented as mean ± SD (n = 8), *P<0.05, **P<0.01, ***P<0.001, ns = not significant. (*d*-*e*) Clonogenic and self-renewal capacity of SC and adherent differentiated counterparts were assessed by performing colony- or sphere-formation assays respectively. 5FU (5μM) was used to assess chemoresistance in both culture types. For T6, 2 independent experiments were performed whereas one single experiment was performed for HT29.

doi:10.1371/journal.pone.0146052.g005

and tumor proliferation underlines and recapitulates the aggressive nature of the primary tumor of patient T20. Six to fifteen weeks after primary transplantation, tumors were explanted, dissociated into single cells and serially transplanted into secondary recipient mice. Injections of 10.000 and 1000 cells allowed the formation of a tumor in secondary recipient mice. Histology assessment of the resulting primary and secondary xenografts by a pathologist confirmed the resemblance to the original primary tumor except that T20 lost some differentiation potential through serial transplantation (Fig 4C). Thus, SC isolated from primary human tumor samples do not only possess tumor-initiating capacity, but they also recapitulate the phenotype of the primary tumor (Fig 4C).

We then thought to directly compare self-renewal capacity of adherent differentiated and spheroid cultures. For this, we assessed the ability for self-renewal in the form of colonies for adherent differentiated cultures and of spheres for SC by plating single cells and counting colonies and spheres, respectively. We could not observe a drastic difference in self-renewal between the two culture types (Fig 5*E* and S5E and S5F Table), although for T6 differentiated cultures were able to form colonies in 55% of the cases (Fig 5*D* and S5E Table) whereas SC generated spheres with 75% efficiency (Fig 5*E*). Adherent differentiated cultures did not form any spheres when maintained in differentiating culture conditions (S4B Fig). However, when these cells were reversed to SCM conditions they were able to form spheres to similar extent, as did cells derived from SC (Fig 5*E* and S4B Fig). We might reason that cells from adherent differentiated cultures may rapidly reacquire a TIC phenotype, suggesting a high plasticity of these

cells. Furthermore, the size of spheres originating from single cells, a measurement of cell proliferation, was determined for both cultures. Strikingly and in accordance with our proliferation assays, spheres derived from differentiated cultures were bigger in size compared to spheres from SC (S3 Fig). In addition we performed colony-forming assays using different cell densities with both culture conditions. The number of colonies did not differ significantly between SC and adherent differentiated counterparts (Fig 5*D* and S5E and S5F Table). Again colonies were bigger in size when derived from differentiated cultures (S3 Fig). Altogether, these data suggest that cultures maintained in SCM or adherent differentiation conditions display similar self-renewal capacity. In addition SC display a weaker proliferation than their adherent counterpart. We further investigated whether passage through xenografts may influence the phenotype of TICs. Xenograft-derived spheres showed similar self-renewal capacity to SC derived from primary tissue or CRC cell lines (S5A and S5B Fig) and for some cases even showed a reduced number of spheres or an altered phenotype (S5C Fig). In order to compare the *in vivo* tumorigenic potential of primary SC and their differentiated adherent counterpart, we injected single cell suspensions of both conditions in NOD/SCID mice. SC showed a tendency for faster tumor growth rate than the differentiated counterpart, which was significant in the early time points (Fig 4*D*). However, tumor incidence was similar between both groups (Fig 4*D*), suggesting that tumor-initiating capacity of TICs is not restricted to phenotypically immature spheroid cells. Of note, resected xenografts derived from SC and differentiated counterparts displayed similar expression patterns of stemness genes (S6C and S6D Fig).

Spheroid cultures are more resistant to chemotherapeutic treatments than adherent differentiated cultures

In order to be able to compare SC and their differentiated counterparts, the same culturing conditions and the same culture medium were applied for both groups and different assays were performed (see Material and Methods section). In the first assay, we decided to use 3D cell culture models to explore the resistance to chemotherapeutics as they have been shown to provide more relevant and translational observations [51]. Cells were plated in round-bottom ULA plates in order to allow the formation of a sphere followed by treatment with 5-FU at different concentrations and sphere size was recorded (Fig 5*A and* 5*B*). The second assay measures the metabolic activity of the cells after 5-FU treatment and thereby reflects their survival rates (Fig 5*C*). Primary SC showed higher resistance to 5-FU treatment and a higher relative survival rate compared to their differentiated counterparts (Fig 5*B and* 5*C*). HCT116 SC and the differentiated counterpart failed to form a unique sphere for the setup of the first assay; therefore, we only included the relative survival assessment for this cell line (Fig 5*C*). Interestingly, in contrast to primary SC, cell line-derived SC display less resistance to 5-FU treatment (S5A–S5D Table), which can also be observed while analyzing the survival rates; in HCT116, no difference was observed in survival rates after 5-FU treatment between SC and differentiated counterpart (Fig 5*C*) and in HT29 the difference between both conditions was less prominent and significant compared to primary SC (Fig 5*C* and S5D Table). As the first assays described are based on sphere size and metabolic activity, which rather reflects cell proliferation, we additionally performed colony and sphere forming assays to assess self-renewal and clonogenic capacity of SC and adherent differentiated cultures. Strikingly, while performing colony forming assays for both culture types, we could observe a drastic reduction in colony formation after 5-FU treatment in adherent differentiated cultures compared to SC (Fig 5*D*). Consistently, sphere-initiating frequency was barely altered after 5-FU treatment in SC whereas a decrease in sphere numbers could be observed for adherent differentiated cultures (Fig 5*E*). Of note, after 5-FU treatment sphere formation was slightly reduced in cells derived from traditional cell

lines (HT29 SC), whereas no difference could be observed for cells derived from primary tumor tissue (T6 SC) (Fig 5*E*). This observation is in line with the results obtained in the two other chemoresistance assays, which might suggest that cell-line derived SC are more sensitive to conventional therapy than SC derived from primary tumor tissue. Different mutational profiles could be the basis for the observed differences in drug response. However, the mutational status of several relevant CRC mutations was identical between SC and their differentiated counterparts and no major differences could be observed between SC derived either from HT29 cell line or from primary tumor tissue (S2 Fig). Taken together, all the described experiments show that SC display increased resistance to chemotherapeutics compared to adherent differentiated cultures. The increased sensitivity to 5-FU is consistent with the high proliferation rate of the adherent cultures, as chemotherapeutics are known to mostly target fast-proliferating cells. SC from primary tumor tissue might therefore represent an interesting tool for *in vitro* studies of new CRC therapies as these culture conditions favor the enrichment of cells that are highly resistant to conventional therapies and that need to be targeted to circumvent disease relapse.

Gene signature derived from spheroid cultures predicts for poor patient outcome in CRC

We could identify a common sphere gene signature consisting of 8 genes that were commonly up-regulated among the primary and cell line-derived SC in comparison to their respective differentiated counterparts (Fig 6*A* and S6 Table). The 8-gene expression signature was able to predict poor overall survival in CRC patients (Fig 6*B*). The clinical relevance of the sphere signature could be confirmed in a second cohort of CRC patients among a collection of datasets available in the PROGgeneV2 tool [41] (S7A Fig). However, as most patients suffering from CRC are already at an advanced age and thus may die from another reason than from CRC, overall survival assessment might not be the adapted measure for patients' outcome. Two genes, namely *CDA* and *GST4A* out of the 8 gene signature are known to be associated with chemoresistance [52–54]. Interestingly, the expression of these genes, either combined or separate, was found to be linked with an increased risk of disease relapse in a large patient cohort covering 565 patients (dataset GSE39582) (Fig 6*C* and S7B and S7C Fig).

Discussion

We first thought to select TICs by using well-described markers. However, while assessing the expression of CD133, CD44 and CD24 in primary tumor CRC tissue and in SC derived from patient material, we observed a very heterogeneous expression. In agreement with our data, several recent studies also showed high variability in the expression of surface markers between different patients, ranging from no or little expression to 90% positivity for the same marker [23,34,48,55]. It has further been demonstrated that culturing conditions (including cell density and passage number) influence surface marker expression to a high degree [56]. Importantly, neither SC derived from patient material nor established cell lines did show any difference in sphere-forming frequency when flow-sorted according to their CD133 expression. Accordingly, over the last years, many reports challenge the view of CD133 as a universal TIC marker [57]. Thus, it seems delicate to rely on surface markers for the isolation of colon TICs.

Another way of identifying TICs is based on their functional characteristics. We established SC in which cells grow in an anchorage-independent manner. In agreement with others [12,19,22,24,30], we provide evidence that SC retain the characteristics of the tumors of origin. A recent study by Lee and colleagues suggests that patient-derived SC have similar mutational and genotype profiles to original CRC tumors [23]. Accordingly, most CRC relevant mutations

Figure 6

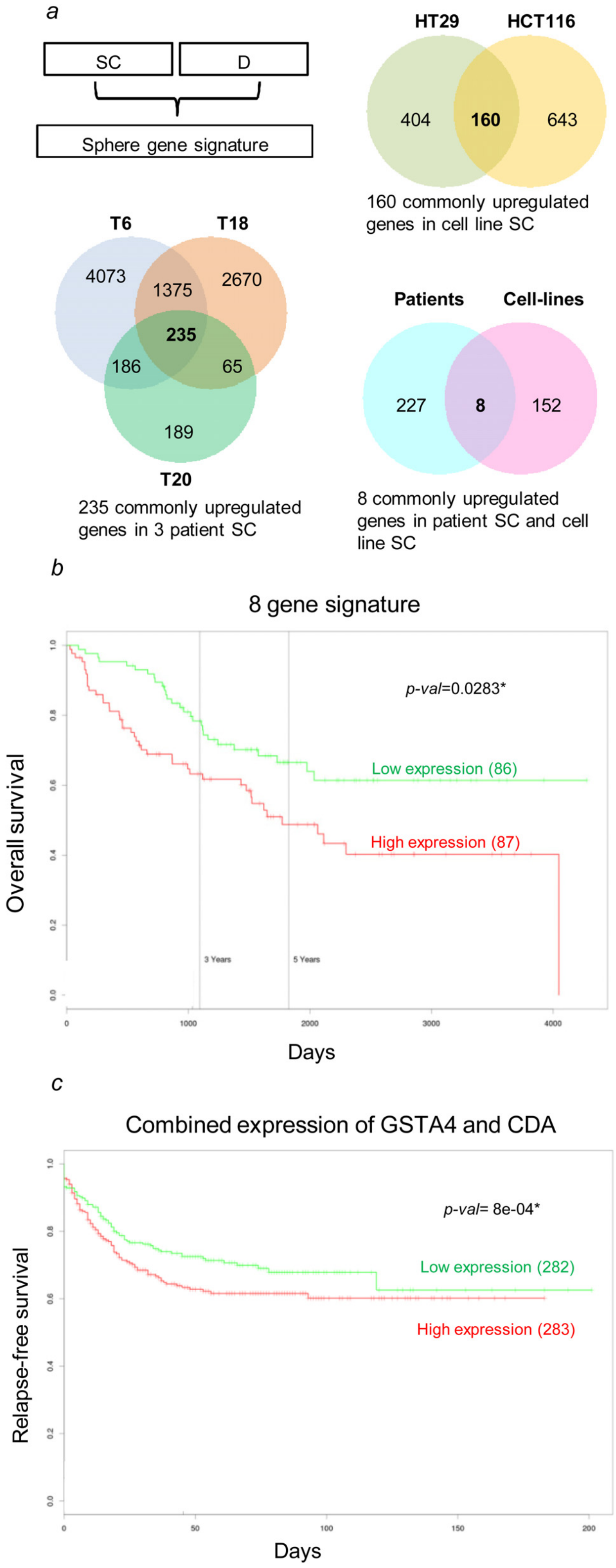
a
SC
D
Sphere gene signature
HT29
HCT116
404
160
643
160 commonly upregulated genes in cell line SC
T6
T18
4073
1375
2670
235
186
65
189
T20
235 commonly upregulated genes in 3 patient SC
Patients
Cell-lines
227
8
152
8 commonly upregulated genes in patient SC and cell line SC
b
8 gene signature
p-val=0.0283*
Low expression (86)
High expression (87)
Overall survival
Days
3 Years
5 Years
c
Combined expression of GSTA4 and CDA
p-val= 8e-04*
Low expression (282)
High expression (283)
Relapse-free survival
Days

Fig 6. Gene signature derived from SC predicts poor outcome in CRC patients. (*a-b*) An overlap of 8 commonly up-regulated genes for patient and cell line-derived SC (listed in S6 Table) predicts for overall survival in CRC. (*b*) The combined expression of two genes, *CDA* & *GSTA4*, is linked to an increased risk of disease relapse. The number of patients in each group is mentioned within the brackets; significant p-value is indicated.

doi:10.1371/journal.pone.0146052.g006

were maintained between the tumor of origin and our generated SC. We extended our study by characterizing these SC in regard to TIC properties. Generated patient-derived SC harbor increased expression of stemness proteins, a property of stem cells and their tumorigenic counterparts [50] and show high self-renewal capacity. Mice transplanted with 100 cells of T6, T18 and T20 all initiated tumor growth, which shows the tumorigenic potential of SC derived from primary tumor tissue. Barrett and colleagues showed that the self-renewal capacity of glioma TICs, determined by the ability of cells to form sphere cultures, is not associated with the tumor growth potential in mouse models; cells with a low self-renewing capacity led to more aggressive tumors *in vivo* compared to higher self-renewing cells [58]. In contrast, we and others [12,59] show that sphere formation correlates to *in vivo* tumorigenicity; the phenotype of T20-derived SC, which is characterized by high SFC frequency, also shows a higher tumor incidence and faster tumor growth, thereby recapitulating the aggressive nature of the primary tumor of patient T20. Nevertheless, this observation needs to be addressed with additional primary SC. In line with Dieter and colleagues we show that long-term passaging of cells under spheroid culture conditions allows for the enrichment in TICs [48]. Importantly, the established primary SC were all capable of tumor initiation at really low cell doses, the gold standard method to evaluate for the presence of TICs.

Interestingly, at the beginning of tumor outgrowth, T20 and HT29 SC show faster tumor growth *in vivo* compared to their differentiated counterpart. However, sphere formation *in vitro* and tumor incidence in immune-deficient mice were similar between cells maintained under spheroid or differentiating culture conditions, suggesting that self-renewal and tumor-initiation capacity is not restricted to phenotypically immature spheroid cells but might instead be attributed to highly proliferative progenitors. These results are in line with other studies comparing differentiated and SC derived from cell lines that showed similar tumorigenic potential for both culturing methods [29,32,35,60]. Our observations agree with the dynamic TIC model that has recently been proposed by Vermeulen and colleagues [15,27]. Indeed, a xenograft might provide a microenvironment that affects plasticity and dedifferentiation processes of tumor cells [61]. Stromal cell-secreted factors have been shown to restore the TIC phenotype by upregulating the Wnt cascade in more differentiated tumor cells both *in vitro* and *in vivo* [27]. Along this line, similar expression of stemness genes was observed in xenografts derived from SC and their differentiated counterpart. Furthermore, we could observe a reversible phenotype when differentiated cultures were exposed to serum-free conditions in presence of growth factors. Altogether, these results suggest a pronounced plasticity of TICs, which needs to be considered for the development of TIC-specific therapies. Further studies will focus on the influence of the microenvironment on the identified plasticity of TICs.

In contrast to hematopoietic malignancies, conventional therapy regimes in solid tumors have only marginally improved the overall survival, illustrating the profound impact of treatment resistance. Thus present therapies, which mostly follow total elimination of rapidly dividing and differentiated tumor cells, need to be modified to target TICs that are able to repopulate the tumor [14]. Compared to their differentiated counterparts, SC showed increased resistance to 5-FU, a conventional chemotherapeutic agent, often used in CRC [62]. Noteworthy, all SC derived from traditional cell lines (HCT116 and HT29) and primary cultures do present the same mutational profile as their respective differentiated counterparts,

suggesting that the higher resistance of SC might not be due to differences in mutations. Along this line, we identified a sphere gene signature that could predict patient outcome in CRC. Interestingly, 7 genes out of the 8-gene signature are known to be involved in tumorigenesis and their function is described in S6 Table. We found two genes, *CDA* and *GSTA4*, to be linked with disease relapse in CRC. Recently, *CDA* was identified as a regulator of cell proliferation and chemoresistance in breast and pancreatic cancer [52,53]. In CRC genomic variations of *CDA* are associated with adverse drug response [63]. Most importantly, high expression of *CDA* was found in blood from CRC patients [64], hinting to a potential use of CDA as a diagnostic marker. *GSTA4* is up-regulated in tumor cells during drug resistance to cisplatin and doxorubicin [54,65,66]. This part of the gene signature nicely supports our findings regarding resistance of our established SC to chemotherapeutics. The obtained differential gene expression in SC relative to their respective differentiated counterparts may partly be a result of the different cell culture media, one containing growth factors (EGF and FGF) and the other not. Despite these differences, genes with clinical relevance in regard to disease recurrence could be identified in a large independent cohort of CRC patients. This finding requires confirmation in additional large cohort studies of CRC patients and the exact role of each gene in TIC function and chemoresistance will be the focus of future investigations. Taken together, we provide evidence that SC derived from primary tumor tissue represent a powerful tool to study the mechanisms of chemotherapy resistance and thereby identify new targets for CRC.

Lately several other groups attempted to isolate TICs from traditional CRC cell lines with conflicting results [20,29,31,32,34,45,60,67–71]. Several studies showed that SC derived from different types of cancer cell lines are more efficient in initiating tumors than adherent monolayers [68,71]. In contrast to these studies, Calvet and colleagues suggest that SC enrich for TICs in a cell line–dependent manner [35]. In their study, sphere formation could not be considered as an efficient method to enrich for TICs in murine melanoma and breast cancer cell lines, although the results were not conclusive for the CRC cell line HT29. Additionally, TICs derived from primary human gliomas have been reported to undergo spontaneous differentiation and apoptosis in SC thereby limiting the stem cell behavior of these cultures compared to adherent culture conditions [72]. One might argue that growth factors access an adherent culture in a more uniform way and thus prevent spontaneous differentiation events enabling expansion of a TIC-enriched population [72]. By performing medium switch experiments, Ahmad and colleagues propose that differences in self-renewal are rather due to the type of medium used and not associated with the fact of the cells being cultured as spheres or adherent cells, supporting the idea that SC are biologically not more relevant in regard to TIC characteristics than monolayers [36]. In the context of CRC cell lines, colon spheres derived from the Caco-2 cell line lost several TIC properties compared to their parental adherent counterpart [32]. Similar observations were made for the HCT116 cell line by Kai and colleagues, where the authors suggested that HCT116-derived SC follow a more stochastic model than a TIC model [60]. Recently, Collura and colleagues performed an extensive characterization of 25 established CRC cell lines and concluded that SC do not seem to present TIC features in regard to tumor-initiating potential but maintain their chemoresistance ability compared to adherent culturing methods [29]. Using sphere formation assays by seeding single cells per well, our SC derived from traditional cell lines showed high self-renewal capacity, which is in line with the expression of stemness proteins. In agreement with the study by Collura and colleagues, we demonstrate that SC-derived cells show increased chemoresistance compared to adherent differentiated cells [29]. In contrast to the study by Ahmad and colleagues, the chemoresistance ability is maintained while cells derived from SC are switched to differentiating culture conditions [36]. These results clearly demonstrate that SC better preserve chemoresistance features of primary cells.

Recent evidence indicates that Wnt signaling activity may serve as a functional designation of TICs [27]. LGR5, a member of the Wnt signaling pathway, has been described as a marker for stem-like cells in CRC [73]. Interestingly, LGR5 is higher expressed in SC derived from primary tumor tissue than from traditional cell lines, hinting to a potential enhanced TIC phenotype in SC derived from primary patient biopsies compared to cell line-derived SC. However, the role of LGR5 in CRC is not yet fully understood. While previous studies suggested that intestinal tumors arise from LGR5-positive cells [74]. Walker and colleagues recently showed that suppression of LGR5 expression enhances tumorigenesis [75]. In our study, cell line-derived SC also show high tumor-initiating capacity similar to primary tumor-derived SC. In contrast to our findings, a recent study by Fan and colleagues compared fresh clinical specimen to established cell lines and concluded that only fresh patient tumor material can be used to isolate TICs [34]. Whereas the authors focus on ALDH activity to isolate TICs we based our study on functional properties of TICs. In future, we foresee to perform cell sorting based on the expression of LGR5 and ALDH followed by functional assays.

In conclusion, we demonstrate that SC derived from primary colon tumor tissue display self-renewal capacity, tumorigenic potential and chemoresistance. SC seem to represent a superior model to adherent differentiated counterparts to screen for new CRC therapies mostly due to their increased resistance to chemotherapeutics. Besides, we show that differentiated cells can rapidly reacquire stem-cell traits which is in line with recent studies demonstrating widespread plasticity and dedifferentiation processes that can affect cancer cells under specific environmental conditions [15,27,61]. Finally, our findings underline the clinical pertinence of our SC, and in future, they may represent an interesting tool to test for new CRC therapies.

Supporting Information

S1 Fig. Fresh patient biopsies are highly heterogeneous in terms of phenotypic markers. **(A)** Flow cytometry analysis of surface markers CD24, CD44 and CD133 in fresh resected tumor tissues from 4 different patients. **(B)** CD133 does not correlate with enhanced sphere-forming capacity. SC were sorted according to low, medium and high expression of the surface marker CD133, followed by a single cell sphere formation assay for CRC cell lines and T6 SC, data are presented as mean ± SD, ns = not significant. Representative figure from 2 independent experiments for HT29 and HCT116 while for T6 one single experiment was performed.
(TIF)

S2 Fig. Mutational profile of the original tumors and established cultures. Several CRC-relevant mutations are shown for primary tumors, primary spheroid (S) and differentiated (D) cultures for T18 and T20 and for differentiated and spheroid cultures derived from cancer cell lines HCT116 and HT29. For ethical purposes we were not able to include the mutational profile for patient T6. Top mutations were assessed using the TruSeq Amplicon-Cancer panel (TSACP), depth > 1000, variant frequency > 0.05. Scale bar represents the number of mutations detected per gene. All mutations found in SC were also present in the differentiated counterpart.
(TIF)

S3 Fig. Differentiated counterparts display a higher proliferative potential compared to SC. **(A)** Cell count observed after 5 days of culture for 100 000 cells initially plated. Representative figure of 2 independent experiments. **(B)** Spheres of differentiated cultures are bigger than spheres derived from SC. **(C)** Colonies derived from differentiated culture T6 are bigger than colonies from T6 SC. For **B-C** data are shown T6 for and HT29 and are representative of 2 independent and 1 single experiment for T6 and HT29, respectively. The number of spheres

analysed for size is indicated on the Fig. Data is presented as mean ± SD, $^{*}P<0.05$, $^{**}P<0.001$, $^{***}P<0.0001$.
(TIF)

S4 Fig. Sphere-forming capacity of established cultures. **(A)** High numbers of cells form spheres just by fusion and aggregation, and not due to increased self-renewal properties. Self-renewal capacity determined by the 1000 cell sphere formation assay of early passage SC grown from primary tumor tissue and CRC cell lines. Number of spheres observed by plating 1000 cells per well does not correlate to the results of the single cell assay presented in Fig 3, which might suggest that fusion and aggregation, rather than self-renewing capacity lead to sphere formation in the 1000 cell assay. Sphere formation was observed over several generations (gen.). Data are presented as mean ± SD. **(B)** Self-renewal capacity determined by the single cell assay of the differentiated counterparts (late passages) reversed to spheroid culturing conditions or maintained in differentiating culturing conditions, respectively. Sphere formation was observed over two generations (gen.). Data is shown for T18 and presented as mean ± SD, $^{*}P<0.05$, $^{**}P<0.001$.
(TIF)

S5 Fig. Sphere-forming capacity of primary and xenograft derived SC. **(A)** and **(B)** Self-renewal capacity, shown by 1000 cell (left panel) and single cell (right panel) sphere forming assays, is not increased in xenograft-derived SC over several generations (gen.) compared to SC directly isolated from fresh tumor tissue. Data are presented as mean of 6 replicates ± SD. Xgen = xenograft-derived generation. **(C)** Xenograft-derived HCT116 SC grow as loosely packed aggregates that no longer resemble to spheres. **(D)** Morphological features of differentiated CRC SC. CRC spheroids adhere and differentiate when grown in medium supplemented with serum.
(TIF)

S6 Fig. *In vivo* tumorigenic potential of SC. **(A)** SC derived from CRC cell lines are able to induce tumors in mice. **(B)** Tumor weight of the generated tumor xenografts. n = 5, data are presented as mean ± SEM. **(C)** and **(D)** SC-derived xenografts (S) and xenografts generated from their differentiated counterparts (D) display similar expression patterns of stemness genes SOX2, OCT4, NANOG and LGR5. Data are presented as mean ± SD, $^{*}P<0.05$, ns = not significant, n = 5.
(TIF)

S7 Fig. Gene signature derived from SC predicts poor outcome in CRC patients. **(A)** Overall survival curves for CRC patients classified according to gene expression levels for the 8-gene sphere signature in the datasets GSE29621. **(B-C)** The expression of two genes from our identified gene signature (CDA & GSTA4) is linked to an increased risk of disease relapse in the dataset GSE39582. The number of patients in each group is mentioned within the brackets; significant p-value is indicated. SC = spheroid cultures, D = differentiated counterparts.
(TIF)

S1 Table. Primary and secondary antibodies used for immunofluorescence analysis and FACS analysis as well as primer sequences used within the study.
(PDF)

S2 Table. Patient Characteristics.
(PDF)

S3 Table. *In vitro* limiting dilution assays of CRC spheroid cultures—Sphere forming cell (SFC) frequency.
(PDF)

S4 Table. *In vivo* limiting dilution assays of CRC spheroid cultures–number of injected cells from dissociated spheroids and tumor formation occurrence in mice.
(PDF)

S5 Table. Chemosensitivity assays in SC and differentiated counterparts. *A*-*D*. Statistics of the sphere formation and 5-FU chemosensitivity assay. 2way ANOVA with Bonferroni post-tests, n = 8, *P<0.05, **P<0.01, ***P<0.001, S = Spheres, D = Differentiated counterpart. ***E*-*F*** Percentages of colony formation in SC and differentiated counterparts after 5FU treatment.
(PDF)

S6 Table. Genes from the 8-gene signature and their functions.
(PDF)

Acknowledgments

The authors would like to thank the contributing surgeons from Centre Hospitalier du Luxembourg and Centre Hospitalier Emile Mayrisch and the nurses of the Clinical and Epidemiological Investigation Center of the Luxembourg Institute of Health (LIH) for work with the patients, as well as the Integrated Biobank of Luxembourg (IBBL), in particular Dr. Fay Betsou and Dr. Nikolai Goncharenko, for the overall set up of the sample collection for the study. The authors thank the pathology team of the IBBL, especially Dr. Laurent Antunes and Dr. Nathalie Marcon for pathological analysis of the primary patient tumors and the serial xenografts. We thank the Genomics Research Unit of LIH headed by Dr. Laurent Vallar, especially Arnaud Muller, for their support with bioinformatics. We thank Brian De Witt from the IBBL for performing the TruSeq Amplicon-Cancer panel assay and we thank Aurélien Ginolhac for the analysis of the mutational profile. We thank Nathalie Nicot of the LIH for performing microarray experiments. We thank Dr. Christelle Bahlawane and Martine Schmitz for informative discussions and critical reading of the manuscript.

Author Contributions

Conceived and designed the experiments: KQB PU SH EL. Performed the experiments: KQB PU FR SF PVN EL. Analyzed the data: KQB PU PVN EL. Contributed reagents/materials/analysis tools: SF PVN. Wrote the paper: KQB EL.

References

1. Siegel R, Naishadham D, Jemal A. Cancer Statistics, 2012. CA Cancer J Clin. 2012; 62:10–29. doi: 10.3322/caac.20138 PMID: 22237781
2. Magee J a, Piskounova E, Morrison SJ. Cancer stem cells: impact, heterogeneity, and uncertainty. Cancer Cell [Internet]. Elsevier Inc.; 2012 [cited 2012 Nov 7]; 21:283–96. Available from: http://www.ncbi.nlm.nih.gov/pubmed/22439924 doi: 10.1016/j.ccr.2012.03.003 PMID: 22439924
3. Kreso A, Dick JE. Evolution of the cancer stem cell model. Cell Stem Cell [Internet]. Elsevier Inc.; 2014; 14:275–91. Available from: http://dx.doi.org/10.1016/j.stem.2014.02.006 doi: 10.1016/j.stem.2014.02.006 PMID: 24607403
4. Al-Hajj M, Wicha MS, Benito-Hernandez A, Morrison SJ, Clarke MF. Prospective identification of tumorigenic breast cancer cells. Proc. Natl. Acad. Sci. U. S. A. 2003; 100:3983–8. PMID: 12629218
5. Dontu G, Abdallah WM, Foley JM, Jackson KW, Clarke MF, Kawamura MJ, et al. In vitro propagation and transcriptional profiling of human mammary stem / progenitor cells. Genes Dev. 2003; 17:1253–70. PMID: 12756227

6. Fang D, Nguyen TK, Leishear K, Finko R, Kulp AN, Hotz S, et al. A tumorigenic subpopulation with stem cell properties in melanomas. Cancer Res. 2005; 65:9328–37. PMID: 16230395
7. Singh S, Hawkins C, Clarke I, Squire J, Bayani J, Hide T, et al. Identification of human brain tumour initiating cells. Nature. 2004; 432:396–401. PMID: 15549107
8. Gilbertson RJ, Rich JN. Making a tumour's bed: glioblastoma stem cells and the vascular niche. Nat. Rev. Cancer. 2007; 7:733–6. PMID: 17882276
9. Hemmati HD, Nakano I, Lazareff J a, Masterman-Smith M, Geschwind DH, Bronner-Fraser M, et al. Cancerous stem cells can arise from pediatric brain tumors. Proc. Natl. Acad. Sci. U. S. A. 2003; 100:15178–83. PMID: 14645703
10. Hermann PC, Huber SL, Herrler T, Aicher A, Ellwart JW, Guba M, et al. Distinct Populations of Cancer Stem Cells Determine Tumor Growth and Metastatic Activity in Human Pancreatic Cancer. Cell Stem Cell. 2007; 1:313–23. doi: 10.1016/j.stem.2007.06.002 PMID: 18371365
11. Bender Kim CF, Jackson EL, Woolfenden AE, Lawrence S, Babar I, Vogel S, et al. Identification of bronchioalveolar stem cells in normal lung and lung cancer. Cell. 2005; 121:823–35. PMID: 15960971
12. Ricci-Vitiani L, Lombardi DG, Pilozzi E, Biffoni M, Todaro M, Peschle C, et al. Identification and expansion of human colon-cancer-initiating cells. Nature [Internet]. 2007 [cited 2012 Oct 25]; 445:111–5. Available from: http://www.ncbi.nlm.nih.gov/pubmed/17122771 PMID: 17122771
13. O'Brien C a, Pollett A, Gallinger S, Dick JE. A human colon cancer cell capable of initiating tumour growth in immunodeficient mice. Nature [Internet]. 2007 [cited 2012 Oct 25]; 445:106–10. Available from: http://www.ncbi.nlm.nih.gov/pubmed/17122772 PMID: 17122772
14. Yang C, Jin K, Tong Y, Cho WC. Therapeutic potential of cancer stem cells. Med. Oncol. [Internet]. 2015 [cited 2015 May 21]; 32:619. Available from: http://www.ncbi.nlm.nih.gov/pubmed/25920610 doi: 10.1007/s12032-015-0619-6 PMID: 25920610
15. Vermeulen L, de Sousa e Melo F, Richel DJ, Medema JP. The developing cancer stem-cell model: clinical challenges and opportunities. Lancet Oncol. [Internet]. Elsevier Ltd; 2012 [cited 2012 Nov 2]; 13: e83–9. Available from: http://www.ncbi.nlm.nih.gov/pubmed/22300863 doi: 10.1016/S1470-2045(11)70257-1 PMID: 22300863
16. Chen K, Huang Y, Chen J. Understanding and targeting cancer stem cells: therapeutic implications and challenges. Acta Pharmacol. Sin. [Internet]. 2013 [cited 2014 Aug 30]; 34:732–40. Available from: http://www.pubmedcentral.nih.gov/articlerender.fcgi?artid=3674516&tool=pmcentrez&rendertype=abstract doi: 10.1038/aps.2013.27 PMID: 23685952
17. Islam F, Gopalan V, Smith RA, Lam AK-Y. Translational potential of cancer stem cells: A review of the detection of cancer stem cells and their roles in Cancer recurrence and cancer treatment. Exp. Cell Res. [Internet]. 2015 [cited 2015 May 12]; Available from: http://www.ncbi.nlm.nih.gov/pubmed/25967525
18. Singh SK, Clarke ID, Terasaki M, Bonn VE, Hawkins C, Squire J, et al. Identification of a cancer stem cell in human brain tumors. Cancer Res. 2003; 63:5821–8. PMID: 14522905
19. Lee J, Kotliarova S, Kotliarov Y, Li A, Su Q, Donin NM, et al. Tumor stem cells derived from glioblastomas cultured in bFGF and EGF more closely mirror the phenotype and genotype of primary tumors than do serum-cultured cell lines. Cancer Cell [Internet]. 2006 [cited 2015 May 26]; 9:391–403. Available from: http://www.ncbi.nlm.nih.gov/pubmed/16697959 PMID: 16697959
20. Ponti D. Isolation and In vitro Propagation of Tumorigenic Breast Cancer Cells with Stem/Progenitor Cell Properties. Cancer Res. 2005; 65:5506–11. PMID: 15994920
21. Todaro M, Alea MP, Di Stefano AB, Cammareri P, Vermeulen L, Iovino F, et al. Colon cancer stem cells dictate tumor growth and resist cell death by production of interleukin-4. Cell Stem Cell [Internet]. 2007 [cited 2012 Mar 8]; 1:389–402. Available from: http://www.ncbi.nlm.nih.gov/pubmed/18371377 doi: 10.1016/j.stem.2007.08.001 PMID: 18371377
22. Vermeulen L, Todaro M, de Sousa Mello F, Sprick MR, Kemper K, Perez Alea M, et al. Single-cell cloning of colon cancer stem cells reveals a multi-lineage differentiation capacity. Proc. Natl. Acad. Sci. U. S. A. [Internet]. 2008; 105:13427–32. Available from: http://www.pubmedcentral.nih.gov/articlerender.fcgi?artid=2533206&tool=pmcentrez&rendertype=abstract doi: 10.1073/pnas.0805706105 PMID: 18765800
23. Lee S-H, Hong JH, Park HK, Park JS, Kim B-K, Lee J-Y, et al. Colorectal cancer-derived tumor spheroids retain the characteristics of original tumors. Cancer Lett. [Internet]. 2015 [cited 2015 Jul 21]; Available from: http://www.ncbi.nlm.nih.gov/pubmed/26185002
24. Kondo J, Endo H, Okuyama H, Ishikawa O, Iishi H, Tsujii M, et al. Retaining cell-cell contact enables preparation and culture of spheroids composed of pure primary cancer cells from colorectal cancer. Proc. Natl. Acad. Sci. U. S. A. [Internet]. 2011 [cited 2012 Nov 8]; 108:6235–40. Available from: http://www.pubmedcentral.nih.gov/articlerender.fcgi?artid=3076886&tool=pmcentrez&rendertype=abstract doi: 10.1073/pnas.1015938108 PMID: 21444794

25. Weiswald L-B, Bellet D, Dangles-Marie V. Spherical Cancer Models in Tumor Biology. Neoplasia [Internet]. Neoplasia Press, Inc.; 2015; 17:1–15. Available from: http://linkinghub.elsevier.com/retrieve/pii/S1476558614001948 doi: 10.1016/j.neo.2014.12.004 PMID: 25622895

26. Hirschhaeuser F, Menne H, Dittfeld C, West J, Mueller-Klieser W, Kunz-Schughart LA. Multicellular tumor spheroids: an underestimated tool is catching up again. J. Biotechnol. [Internet]. 2010 [cited 2014 Jul 16]; 148:3–15. Available from: http://www.ncbi.nlm.nih.gov/pubmed/20097238 doi: 10.1016/j.jbiotec.2010.01.012 PMID: 20097238

27. Vermeulen L, De Sousa E Melo F, van der Heijden M, Cameron K, de Jong JH, Borovski T, et al. Wnt activity defines colon cancer stem cells and is regulated by the microenvironment. Nat. Cell Biol. [Internet]. Nature Publishing Group; 2010 [cited 2012 Oct 25]; 12:468–76. Available from: http://www.nature.com/doifinder/10.1038/ncb2048 doi: 10.1038/ncb2048 PMID: 20418870

28. Cammareri P, Lombardo Y, Francipane MG, Bonventre S, Todaro M, Stassi G. Isolation and culture of colon cancer stem cells. Methods Cell Biol. [Internet]. 2008 [cited 2011 Dec 13]; 86:311–24. Available from: http://www.ncbi.nlm.nih.gov/pubmed/18442654 doi: 10.1016/S0091-679X(08)00014-9 PMID: 18442654

29. Collura A, Marisa L, Trojan D, Buhard O, Lagrange A, Saget A, et al. Extensive characterization of sphere models established from colorectal cancer cell lines. Cell. Mol. Life Sci. [Internet]. 2013 [cited 2013 Nov 11]; 70:729–42. Available from: http://www.ncbi.nlm.nih.gov/pubmed/23007843 doi: 10.1007/s00018-012-1160-9 PMID: 23007843

30. Weiswald L-B, Richon S, Massonnet G, Guinebretière J-M, Vacher S, Laurendeau I, et al. A short-term colorectal cancer sphere culture as a relevant tool for human cancer biology investigation. Br. J. Cancer [Internet]. 2013 [cited 2015 Nov 18]; 108:1720–31. Available from: http://www.pubmedcentral.nih.gov/articlerender.fcgi?artid=3668460&tool=pmcentrez&rendertype=abstract doi: 10.1038/bjc.2013.132 PMID: 23538387

31. Muraro MG, Mele V, Daster S, Han J, Heberer M, Cesare Spagnoli G, et al. CD133+, CD166+CD44+, and CD24+CD44+ Phenotypes Fail to Reliably Identify Cell Populations with Cancer Stem Cell Functional Features in Established Human Colorectal Cancer Cell Lines. Stem Cells Transl. Med. 2012; 1:592–603. doi: 10.5966/sctm.2012-0003 PMID: 23197865

32. Wu H, Zhang H, Hu Y, Xia Q, Liu C, Li Y, et al. Sphere formation assay is not an effective method for cancer stem cell derivation and characterization from the Caco-2 colorectal cell line. Curr. Stem Cell Res. Ther. [Internet]. 2014 [cited 2015 Apr 22]; 9:82–8. Available from: http://www.ncbi.nlm.nih.gov/pubmed/24359140 PMID: 24359140

33. Kai K, Nagano O, Sugihara E, Arima Y, Sampetrean O, Ishimoto T, et al. Maintenance of HCT116 colon cancer cell line conforms to a stochastic model but not a cancer stem cell model. Cancer Sci. [Internet]. 2009 [cited 2012 Nov 15]; 100:2275–82. Available from: http://www.ncbi.nlm.nih.gov/pubmed/19737148 doi: 10.1111/j.1349-7006.2009.01318.x PMID: 19737148

34. Fan F, Bellister S, Lu J, Ye X, Boulbes DR, Tozzi F, et al. The requirement for freshly isolated human colorectal cancer (CRC) cells in isolating CRC stem cells. Br. J. Cancer [Internet]. 2014; 112:539–46. Available from: http://www.nature.com/doifinder/10.1038/bjc.2014.620 doi: 10.1038/bjc.2014.620 PMID: 25535733

35. Calvet CY, André FM, Mir LM. The culture of cancer cell lines as tumorspheres does not systematically result in cancer stem cell enrichment. PLoS One [Internet]. 2014 [cited 2015 Jul 13]; 9:e89644. Available from: http://www.pubmedcentral.nih.gov/articlerender.fcgi?artid=3933663&tool=pmcentrez&rendertype=abstract doi: 10.1371/journal.pone.0089644 PMID: 24586931

36. Ahmad M, Frei K, Willscher E, Stefanski A, Kaulich K, Roth P, et al. How Stemlike Are Sphere Cultures From Long-term Cancer Cell Lines ? Lessons From Mouse Glioma Models. 2014; 73:1062–77.

37. Williams S a, Anderson WC, Santaguida MT, Dylla SJ. Patient-derived xenografts, the cancer stem cell paradigm, and cancer pathobiology in the 21st century. Lab. Invest. [Internet]. Nature Publishing Group; 2013; 93:970–82. Available from: http://www.ncbi.nlm.nih.gov/pubmed/23917877 doi: 10.1038/labinvest.2013.92 PMID: 23917877

38. Hu Y, Smyth GK. ELDA: extreme limiting dilution analysis for comparing depleted and enriched populations in stem cell and other assays. J. Immunol. Methods [Internet]. 2009; 347:70–8. Available from: http://www.ncbi.nlm.nih.gov/pubmed/19567251 doi: 10.1016/j.jim.2009.06.008 PMID: 19567251

39. Vandesompele J, De Preter K, Pattyn F, Poppe B, Van Roy N, De Paepe A, et al. Accurate normalization of real-time quantitative RT-PCR data by geometric averaging of multiple internal control genes. Genome Biol. [Internet]. 2002; 3:RESEARCH0034. Available from: http://www.pubmedcentral.nih.gov/articlerender.fcgi?artid=126239&tool=pmcentrez&rendertype=abstract PMID: 12184808

40. Hellemans J, Mortier G, De Paepe A, Speleman F, Vandesompele J. qBase relative quantification framework and software for management and automated analysis of real-time quantitative PCR data. Genome Biol. [Internet]. 2007 [cited 2014 Jul 9]; 8:R19. Available from: http://www.pubmedcentral.nih.gov/articlerender.fcgi?artid=1852402&tool=pmcentrez&rendertype=abstract PMID: 17291332

41. Goswami CP, Nakshatri H. PROGgene: gene expression based survival analysis web application for multiple cancers. J. Clin. Bioinforma. [Internet]. Journal of Clinical Bioinformatics; 2013; 3:22. Available from: http://www.jclinbioinformatics.com/content/3/1/22 doi: 10.1186/2043-9113-3-22 PMID: 24165311

42. Smith JJ, Deane NG, Wu F, Merchant NB, Zhang B, Jiang A, et al. Experimentally derived metastasis gene expression profile predicts recurrence and death in patients with colon cancer. Gastroenterology. 2010; 138:958–68. doi: 10.1053/j.gastro.2009.11.005 PMID: 19914252

43. Chen DT, Hernandez JM, Shibata D, Mccarthy SM, Humphries LA, Clark W, et al. Complementary strand microRNAs mediate acquisition of metastatic potential in colonic adenocarcinoma. J. Gastrointest. Surg. 2012; 16:905–13. doi: 10.1007/s11605-011-1815-0 PMID: 22362069

44. Marisa L, de Reyniès A, Duval A, Selves J, Gaub MP, Vescovo L, et al. Gene expression classification of colon cancer into molecular subtypes: characterization, validation, and prognostic value. PLoS Med. [Internet]. 2013 [cited 2013 Nov 9]; 10:e1001453. Available from: http://www.pubmedcentral.nih.gov/articlerender.fcgi?artid=3660251&tool=pmcentrez&rendertype=abstract doi: 10.1371/journal.pmed.1001453 PMID: 23700391

45. Ieta K, Tanaka F, Haraguchi N, Kita Y, Sakashita H, Mimori K, et al. Biological and genetic characteristics of tumor-initiating cells in colon cancer. Ann. Surg. Oncol. 2008; 15:638–48. PMID: 17932721

46. Shmelkov S V, Butler JM, Hooper AT, Hormigo A, Kushner J, Milde T, et al. CD133 expression is not restricted to stem cells, and both CD133+ and CD133- metastatic colon cancer cells initiate tumors. J. Clin. Invest. [Internet]. 2008 [cited 2015 Jul 11]; 118:2111–20. Available from: http://www.pubmedcentral.nih.gov/articlerender.fcgi?artid=2391278&tool=pmcentrez&rendertype=abstract doi: 10.1172/JCI34401 PMID: 18497886

47. Dangles-Marie V, Pocard M, Richon S, Weiswald L-B, Assayag F, Saulnier P, et al. Establishment of human colon cancer cell lines from fresh tumors versus xenografts: comparison of success rate and cell line features. Cancer Res. [Internet]. 2007 [cited 2012 Nov 6]; 67:398–407. Available from: http://www.ncbi.nlm.nih.gov/pubmed/17210723 PMID: 17210723

48. Dieter SM, Ball CR, Hoffmann CM, Nowrouzi A, Herbst F, Zavidij O, et al. Distinct types of tumor-initiating cells form human colon cancer tumors and metastases. Cell Stem Cell [Internet]. 2011 [cited 2012 Nov 8]; 9:357–65. Available from: http://www.ncbi.nlm.nih.gov/pubmed/21982235 doi: 10.1016/j.stem.2011.08.010 PMID: 21982235

49. Yamanaka S. Induction of pluripotent stem cells from mouse fibroblasts by. Cell Prolif. 2008; 41:51–6. doi: 10.1111/j.1365-2184.2008.00493.x PMID: 18181945

50. Liu A, Yu X, Liu S. Pluripotency transcription factors and cancer stem cells: Small genes make a big difference. Chin. J. Cancer. 2013; 32:483–7. doi: 10.5732/cjc.012.10282 PMID: 23419197

51. Vinci M, Gowan S, Boxall F, Patterson L, Zimmermann M, Court W, et al. Advances in establishment and analysis of three-dimensional tumor spheroid-based functional assays for target validation and drug evaluation. BMC Biol. [Internet]. BioMed Central Ltd; 2012; 10:29. Available from: http://www.biomedcentral.com/1741-7007/10/29 doi: 10.1186/1741-7007-10-29 PMID: 22439642

52. Ye F-G, Song C-G, Cao Z-G, Xia C, Chen D-N, Chen L, et al. Cytidine Deaminase Axis Modulated by miR-484 Differentially Regulates Cell Proliferation and Chemoresistance in Breast Cancer. Cancer Res. [Internet]. 2015 [cited 2015 Apr 29]; 75:1504–15. Available from: http://cancerres.aacrjournals.org/content/75/7/1504.long doi: 10.1158/0008-5472.CAN-14-2341 PMID: 25643696

53. Weizman N, Krelin Y, Shabtay-Orbach A, Amit M, Binenbaum Y, Wong RJ, et al. Macrophages mediate gemcitabine resistance of pancreatic adenocarcinoma by upregulating cytidine deaminase. Oncogene [Internet]. 2014 [cited 2015 Feb 13]; 33:3812–9. Available from: http://www.ncbi.nlm.nih.gov/pubmed/23995783 doi: 10.1038/onc.2013.357 PMID: 23995783

54. Kalinina E V, Berozov TT, Shtil AA, Chernov NN, Glasunova VA, Novichkova MD, et al. Expression of Genes of Glutathione Transferase Isoforms GSTP1-1, GSTA4-4, and GSTK1-1 in Tumor Cells during the Formation of Drug Resistance to Cisplatin. 2012; 154:64–7. PMID: 23330092

55. Miranda-Lorenzo Irene; Dorado Jorge; Lonardo Enza; Alcala Sonia; Serrano Alicia G.; Clausell-Tormos Jenifer; Cioffi Michele; Megias Diego; Zagorac Sladjana; Balic Anamaria; Hidalgo Manuel; Erkan Mert; Kleeff Joerg; Scarpa Aldo; Sainz Br C Jr.. Intracellular auto-fluorescence–A novel and universally inherited biomarker in epithelial cancer stem cells. Nat. Methods. 2014; 11.

56. Sgambato a, Puglisi M a, Errico F, Rafanelli F, Boninsegna a, Rettino a, et al. Post-translational modulation of CD133 expression during sodium butyrate-induced differentiation of HT29 human colon cancer cells: implications for its detection. J. Cell. Physiol. [Internet]. 2010 [cited 2012 Nov 19]; 224:234–41. Available from: http://www.ncbi.nlm.nih.gov/pubmed/20333645 doi: 10.1002/jcp.22124 PMID: 20333645

57. Kemper K, Grandela C, Medema JP. Molecular identification and targeting of colorectal cancer stem cells. Oncotarget [Internet]. 2010 [cited 2015 May 29]; 1:387–95. Available from: http://www.

pubmedcentral.nih.gov/articlerender.fcgi?artid=3248116&tool=pmcentrez&rendertype=abstract PMID: 21311095

58. Barrett LE, Granot Z, Coker C, Iavarone A, Hambardzumyan D, Holland EC, et al. Self-renewal does not predict tumor growth potential in mouse models of high-grade glioma. Cancer Cell [Internet]. Elsevier Inc.; 2012; 21:11–24. Available from: http://dx.doi.org/10.1016/j.ccr.2011.11.025 doi: 10.1016/j.ccr.2011.11.025 PMID: 22264785

59. O'Brien C a, Kreso A, Ryan P, Hermans KG, Gibson L, Wang Y, et al. ID1 and ID3 regulate the self-renewal capacity of human colon cancer-initiating cells through p21. Cancer Cell [Internet]. Elsevier Inc.; 2012 [cited 2013 Feb 28]; 21:777–92. Available from: http://www.ncbi.nlm.nih.gov/pubmed/22698403 doi: 10.1016/j.ccr.2012.04.036 PMID: 22698403

60. Kai K, Nagano O, Sugihara E, Arima Y, Sampetrean O, Ishimoto T, et al. Maintenance of HCT116 colon cancer cell line conforms to a stochastic model but not a cancer stem cell model. Cancer Sci. [Internet]. 2009 [cited 2012 Mar 8]; 100:2275–82. Available from: http://www.ncbi.nlm.nih.gov/pubmed/19737148 doi: 10.1111/j.1349-7006.2009.01318.x PMID: 19737148

61. Medema JP, Vermeulen L. Microenvironmental regulation of stem cells in intestinal homeostasis and cancer. Nature [Internet]. 2011 [cited 2012 Oct 25]; 474:318–26. Available from: http://www.ncbi.nlm.nih.gov/pubmed/21677748 doi: 10.1038/nature10212 PMID: 21677748

62. Rödel C, Liersch T, Becker H, Fietkau R, Hohenberger W, Hothorn T, et al. Preoperative chemoradiotherapy and postoperative chemotherapy with fluorouracil and oxaliplatin versus fluorouracil alone in locally advanced rectal cancer: Initial results of the German CAO/ARO/AIO-04 randomised phase 3 trial. Lancet Oncol. 2012; 13:679–87. doi: 10.1016/S1470-2045(12)70187-0 PMID: 22627104

63. García-González X, Cortejoso L, García MI, García-Alfonso P, Robles L, Grávalos C, et al. Variants in CDA and ABCB1 are predictors of capecitabine-related adverse reactions in colorectal cancer. Oncotarget [Internet]. 2015 [cited 2015 Apr 29]; 6:6422–30. Available from: http://www.ncbi.nlm.nih.gov/pubmed/25691056 PMID: 25691056

64. Han M, Choong TL, Hong WZ, Chao S, Zheng R, Kok TY, et al. Novel blood-based, five-gene biomarker set for the detection of colorectal cancer. Clin. Cancer Res. 2008; 14:455–60. doi: 10.1158/1078-0432.CCR-07-1801 PMID: 18203981

65. Kalinina E V, Chernov NN, Saprin AN, Kotova YN, Remizov VI, Shcherbak NP. Expression of genes for redox-dependent glutathione S-transferase isoforms GSTP1-1 and GSTA4-4 in tumor cell during the development doxorubicin resistance. Bull. Exp. Biol. Med. [Internet]. 2007 [cited 2015 Aug 6]; 143:328–30. Available from: http://www.ncbi.nlm.nih.gov/pubmed/18225754 PMID: 18225754

66. He NG, Singhal SS, Srivastava SK, Zimniak P, Awasthi YC, Awasthi S. Transfection of a 4-hydroxynonenal metabolizing glutathione S-transferase isozyme, mouse GSTA4-4, confers doxorubicin resistance to Chinese hamster ovary cells. Arch. Biochem. Biophys. [Internet]. 1996 [cited 2015 Aug 6]; 333:214–20. Available from: http://www.ncbi.nlm.nih.gov/pubmed/8806773 PMID: 8806773

67. Ohata H, Ishiguro T, Aihara Y, Sato A, Sakai H, Sekine S, et al. Induction of the Stem-like Cell Regulator CD44 by Rho Kinase Inhibition Contributes to the Maintenance of Colon Cancer-Initiating Cells. Cancer Res. [Internet]. 2012 [cited 2012 Nov 8]; 72:5101–10. Available from: http://www.ncbi.nlm.nih.gov/pubmed/23002207 doi: 10.1158/0008-5472.CAN-11-3812 PMID: 23002207

68. Zhao R, Quaroni L, Casson AG. Identification and characterization of stemlike cells in human esophageal adenocarcinoma and normal epithelial cell lines. J. Thorac. Cardiovasc. Surg. [Internet]. 2012 [cited 2015 Nov 18]; 144:1192–9. Available from: http://www.sciencedirect.com/science/article/pii/S0022522312009567 doi: 10.1016/j.jtcvs.2012.08.008 PMID: 22980068

69. Li L, Ying J, Li H, Zhang Y, Shu X, Fan Y, et al. The human cadherin 11 is a pro-apoptotic tumor suppressor modulating cell stemness through Wnt/β-catenin signaling and silenced in common carcinomas. Oncogene [Internet]. 2012 [cited 2012 Nov 19]; 31:3901–12. Available from: http://www.pubmedcentral.nih.gov/articlerender.fcgi?artid=3426851&tool=pmcentrez&rendertype=abstract doi: 10.1038/onc.2011.541 PMID: 22139084

70. Zhang G, Ma L, Xie Y-K, Miao X-B, Jin C. Esophageal cancer tumorspheres involve cancer stem-like populations with elevated aldehyde dehydrogenase enzymatic activity. Mol. Med. Rep. [Internet]. 2012 [cited 2015 Nov 18]; 6:519–24. Available from: http://www.ncbi.nlm.nih.gov/pubmed/22684859 doi: 10.3892/mmr.2012.939 PMID: 22684859

71. Qiu X, Wang Z, Li Y, Miao Y, Ren Y, Luan Y. Characterization of sphere-forming cells with stem-like properties from the small cell lung cancer cell line H446. Cancer Lett. [Internet]. 2012 [cited 2015 Nov 18]; 323:161–70. Available from: http://www.ncbi.nlm.nih.gov/pubmed/22521544 doi: 10.1016/j.canlet.2012.04.004 PMID: 22521544

72. Pollard SM, Yoshikawa K, Clarke ID, Danovi D, Stricker S, Russell R, et al. Glioma Stem Cell Lines Expanded in Adherent Culture Have Tumor-Specific Phenotypes and Are Suitable for Chemical and Genetic Screens. Cell Stem Cell [Internet]. Elsevier Ltd; 2009; 4:568–80. Available from: http://dx.doi.org/10.1016/j.stem.2009.03.014 doi: 10.1016/j.stem.2009.03.014 PMID: 19497285

73. Hirsch D, Barker N, Mcneil N, Hu Y, Camps J, Mckinnon K, et al. LGR5 positivity defines stem-like cells in colorectal cancer. Carcinogenesis. 2014; 35:849–58. doi: 10.1093/carcin/bgt377 PMID: 24282287

74. Barker N, Ridgway R a, van Es JH, van de Wetering M, Begthel H, van den Born M, et al. Crypt stem cells as the cells-of-origin of intestinal cancer. Nature [Internet]. 2009 [cited 2012 Oct 25]; 457:608–11. Available from: http://www.ncbi.nlm.nih.gov/pubmed/19092804 doi: 10.1038/nature07602 PMID: 19092804

75. Walker F, Zhang H-H, Odorizzi A, Burgess AW. LGR5 is a negative regulator of tumourigenicity, antagonizes Wnt signalling and regulates cell adhesion in colorectal cancer cell lines. PLoS One [Internet]. 2011 [cited 2015 Jul 21]; 6:e22733. Available from: http://www.pubmedcentral.nih.gov/articlerender.fcgi?artid=3145754&tool=pmcentrez&rendertype=abstract doi: 10.1371/journal.pone.0022733 PMID: 21829496
